# Understanding the Effects of Self-Induced Anaerobic Fermentation on Coffee Beans Quality: Microbiological, Metabolic, and Sensory Studies

**DOI:** 10.3390/foods12010037

**Published:** 2022-12-22

**Authors:** Alexander da Silva Vale, Gabriel Balla, Luiz Roberto Saldanha Rodrigues, Dão Pedro de Carvalho Neto, Carlos Ricardo Soccol, Gilberto Vinícius de Melo Pereira

**Affiliations:** 1 Department of Bioprocess Engineering and Biotechnology, Federal University of Paraná (UFPR), Curitiba 81531-980, PR, Brazil; 2 Federal Institute of Education, Science and Technology of Paraná (IFPR), Londrina 86041-120, PR, Brazil

**Keywords:** post-harvest, coffee fermentation, specialty coffee, sensory analysis

## Abstract

In this study, an investigation of the microbial community structure and chemical changes in different layers of a static coffee beans fermentation tank (named self-induced anaerobic fermentation—SIAF) was conducted at different times (24, 48, and 72 h). The microbial taxonomic composition comprised a high prevalence of Enterobacteriaceae and Nectriaceae and low prevalence of lactic acid bacteria and yeast, which greatly differs from the traditional process performed in open tanks. No major variation in bacterial and fungal diversity was observed between the bottom, middle, and top layers of the fermentation tank. On the other hand, the metabolism of these microorganisms varied significantly, showing a higher consumption of pulp sugar and production of metabolites in the bottom and middle layers compared to the top part of the fermentation tank. Extended processes (48 and 72 h) allowed a higher production of key-metabolites during fermentation (e.g., 3-octanol, ethyl acetate, and amyl acetate), accumulation in roasted coffee beans (acetic acid, pyrazine, methyl, 2-propanone, 1-hydroxy), and diversification of sensory profiles of coffee beverages compared to 24 h of fermentation process. In summary, this study demonstrated that SIAF harbored radically different dominant microbial groups compared to traditional coffee processing, and diversification of fermentation time could be an important tool to provide coffee beverages with novel and desirable flavor profiles.

## 1. Introduction

The sharp rise in the specialty coffee market has been partly driven by the continuous growth of out-of-home consumption in Europe, the United States, Brazil, and Australia [1,2,3]. To reach this market, companies have intensified research to diversify the sensory profile of coffee beverages. The influence of the coffee tree genotype, the climate of the producing region (precipitation, humidity, temperature, and radiation), edaphic factors (acidity/alkalinity and soil fertility), and postharvest processing has been widely studied [4,5,6,7]. The combination and interaction of these factors are unique to each region, and small changes have a major impact on the sensory profile of the final beverage [8,9].

In recent years, new post-harvest processing methods have been developed to modulate the chemical structure of coffee beans. The greatest diversification has occurred in the coffee bean fermentation process, in which the natural sugars in the coffee cherry are converted into alcohol, lactic acid, and a range of other secondary metabolites [6,10]. Although this process is often associated with coffee bean mucilage removal, several studies have shown that microbial metabolites diffuse into the beans and alter the quality of the final beverage [11,12,13,14]. Carvalho Neto et al. [15,16] recently proposed the use of a stirred-tank reactor (STR) and starter culture to standardize coffee fermentation, which is usually carried out spontaneously in open tanks. Fermentations conducted in STR were able to improve coffee beverage quality by about 8 points (82.25–91.5) when compared to the traditional process. This increase was associated with temperature and agitation control enabled by the STR method. However, a techno-economic analysis conducted by Magalhães Júnior et al. [17] showed that, for STR implementation, an investment of about USD 1.4 milion is required, which limits the access of this technology to small coffee producers. 

Recently, a new low-cost fermentation method (called self-induced anaerobiosis fer-mentation—SIAF) performed in a cylindrical polyethylene tank has been proposed as an alternative for coffee growers [18]. Several processing configurations have been evaluated. For example, the so-called “natural” fermentation method, which consists in fermenting the whole fruit (i.e., without pulping) or with pulped fruit in static and closed fermentation tanks, without [19,20] or with [21] the use of water. Regardless of the configuration used, this new processing method has resulted in a significant improvement in the final beverage when compared to conventional processing. It is speculated that the anaerobic environment can positively modulate the fermentative performance of the indigenous microbiota, as well as increase the production of desired compounds, such as lactic acid, esters, aldehydes, and ketones. However, the microbial succession of fermentations performed by the SIAF method has been little explored, and dedicated studies have characterized bacterial and fungal communities only at the end of the fermentation process [19,20,22]. Therefore, this work aimed to understand the microbial ecology and metabolites formed during standard (24 h) and extended (48 and 72 h) fermentation in the bottom, middle, and top layers of a static fermentation tank. Finally, sensory analysis was performed to determine the impact of processing time on the quality of the final beverage.

## 2. Materials and Methods

### 2.1. Coffee Fermentation Process

**Figure 1:**
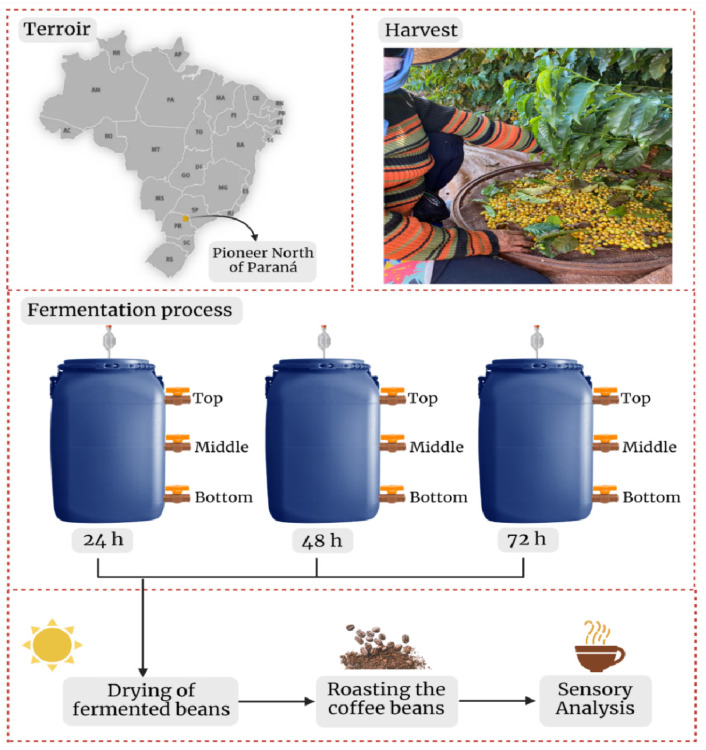
Flowchart showing the steps performed from harvest to sensory analysis.

Fermentations were conducted at California farm (23°08′58.42″ S 50°01′39.33″ W; 650 m above sea level) located in Pioneer North of Paraná (Jacarezinho, Paraná, Brazil). Coffee cherries (*Coffea arabica* var. Catuaí Amarelo IAC 86) were harvested in July 2021 and mechanically pulped. About 175 kg of the pulped coffee beans were transferred to polypropylene fermentation tanks containing 30 L of water previously decontaminated with ozone. These fermentation tanks were cylindrical, 82 cm in height, and 47 cm in diameter. In addition, the fermentation tank contained three taps to perform the sample collection in the bottom, middle, and top layers (i.e., 15, 44, and 72 cm from the base of the fermentation tank, respectively), as shown in (Figure 1). The bioreactors were closed with a screw cap containing an airlock to allow the CO_2_ generated during fermentation to escape and to prevent air from entering the bioreactor (self-induced anaerobioses fermentation—SIAF). Then, a standard fermentation of 24 h and two extended fermentations of 48 and 72 h were performed. The experiments were conducted in triplicate and about 50 mL of the fermentation liquid fraction was collected at 0, 6, 12, 18, 24, 30, 36, 48, 60, and 72 h for microbiological and physicochemical analyses. After the fermentations, coffee beans were washed and dispersed in a raised beds for drying until they reached a moisture content of approximately 12%. 

### 2.2. Determination of Microbial Diversity by High-Throughput Sequencing

DNA was extracted from samples collected at 0, 12, 24, 36, 48, and 72 h from the bottom, middle, and top layers using the Power Soil Kit (Qiagen, Carlsbad, CA, USA). DNA was quantified with a Nanodrop spectrophotometer (Thermo Fisher Scientific, Waltham, MA, USA). The variable regions V3-V4 of the 16S rRNA gene of bacteria were amplified from the extracted total DNA using the primers 341F (TCGTCGGCAGCGTCAGATGTGTATAAGACAGAGCCTACGGGNGGCWGCAG), and 805R (GTCTCGTGGGCTCGGAGATGTGTATAAGAGACAGGACTACHVGGG-TATCTAATCC), while for amplification of the ITS region of the fungi, primers 3F (TCGTCGGCAGCGTCAGATGTGTATAAGACAGGCATCGATGAAGAAC-GCAGC) and 805R (GTCTCGTGGCTCGGAGATGTGTATAAGACAG-TCCTCCGCTTATTGATGC) were used, and both were barcoded with Nextera indices, according to the manufacturer’s instructions (Illumina Inc., San Diego, CA, USA). The amplicons were quantified with the Qubit DNA HS kit (Thermo) and sequenced with the MiSeq Reagent 500 v2 kit (Illumina), in paired 2 x250 b. The fastq files were filtered and demultiplexed with bcl2fastq (Illumina). Taxonomic identification was performed with QIIME2 software package, version 1.9.0. Shannon, Simpson, and Chao indices were calculated and used to determine the alpha and beta diversity of the microbial communities. 

### 2.3. Analysis of Sugar Consumption and Organic Acid Production in the Liquid Fermentation Fraction

High-Performance Liquid Chromatography (HPLC) was used to determine the concentration of sugars (glucose and fructose) and organic acids (lactic, acetic, malic, succinic, citric, and propionic acids) from the bottom, middle, and top layers of fermentation tanks. Aliquots of 2 mL were centrifuged at 12,000× rpm for 10 min and filtered through a 0.22 µm pore size filter (Millipore Corp., Billerica, MA, USA). The samples were analyzed in an HPLC (Agilent Technologies, Waldbronn, Germany) coupled to a diode matrix (DAD) and refraction index (RID). The separation of the compounds was obtained using a Hiplex-H column (300 × 7.7 mm) (Bio-Rad, Richmond, CA, USA) with an isocratic mobile phase composed of 4.0 mM H2SO4, with a flow rate of 0.5 mL min^−1^ for 30 min. The temperatures of the column and RID detector used during the entire race were 70 and 50 °C, respectively. The quantification of organic acids was performed in DAD at 210 nm, while reducing sugars were determined in RID [23]. 

### 2.4. GC/MS Analysis of the Fermentation Liquid Fraction

The volatile organic compounds generated during fermentation were identified by Gas Chromatography Coupled to Mass Spectrophotometry (GC/MS). For sample preparation, aliquots (3 mL) collected from the bottom, middle, and top layers at 0, 12, 24, 48, and 72 h were disposed of in hermetically sealed vials (20 mL). The samples were analyzed by Solid Phase Microextraction (SPME), using a DVB/CAR/PDMS Fiber (Supelco Co., Bellefonte, PA, USA). The SPME fiber was exposed for 30 min at 60 °C. The compounds were thermally desorbed at 260 °C and directly introduced into the gas chromatograph. The GC was equipped with a capillary column (model SH-Rtx-5MS; 30 m × 0.25 mm × 0.25 µm). The temperature within the GC was as follows: column oven at 60 °C, injection at 260 °C, and detector at 250 °C. Helium was the carrier gas used, at a flow rate of 1 mL/min, column press of 57.4 kPa, and split ratio of 1:20. The mass spectrophotometry range was 30–250 (*m*/*z*), at an ion source temperature of 250 °C. Volatiles were identified by comparing each mass spectrum either with the spectra from authentic compounds or with spectra in reference libraries. The relative abundance of each volatile compound present in the headspace was shown as peak area times 10^5^ [24].

### 2.5. GC/MS Analysis of Green and Roasted Coffee Beans

The volatile profile of green coffee beans (i.e., fermented and dried coffee beans) processed for 24, 48, and 72 h was also determined by GC/MS. Briefly, beans with approximately 12% moisture were ground and the particles were standardized with a 0.35 mm sieve. Then, 3 g of the ground coffee was transferred into a hermetically sealed vial (20 mL). 

To perform the analysis of the roasted beans, a roasting curve was developed to preserve the cellular structure of the beans using a Probat Leogap equipment model Probatino (Curtiba, Brazil). A lower initial temperature (130 °C) was used for this process, followed by a gradual increase in temperature, allowing for a longer roast (10:30 min) and a 17.4% development period (1:50 min after the first crack). A Javalytics handheld digital colorimeter model JAV-RDA-H was used 2 h after roasting to check the roast level according to the Specialty Coffee Association (SCA) protocol of 63 Agtron (see https://sca.coffee/research/coffee-standards, accessed on 10 November 2021). Then, the roasted beans were also ground, and the particles were standardized to 0.35 mm and transferred to the vial. The injection parameters used for the green and roasted beans followed the procedures described in the previous topic.

### 2.6. Sensory Analysis

To determine the effect of time on final beverage quality, roasted beans processed for 24, 48, and 72 h were prepared according to SCA recommendations (https://www.scaa.org/PDF/resources/cupping-protocols.pdf, accessed on 15 November 2021). Five cups containing 8.25 g of coffee and 150 mL of water were prepared for each sample. Then two certified Q-Graders were asked to describe and score the attributes aroma, flavor, aftertaste, acidity, body, balance, uniformity, clean cup, sweetness, and overall quality on a scale of 6 to 10 in intervals of 0.25. To facilitate the visualization of the sensory profile of each coffee, a sensory wheel was built in the Tastify^®^ application (https://www.tastify.com/, accessed on 20 July 2022). 

### 2.7. Statistical Analysis

The data obtained from the analyses of sugar consumption and production of volatile organic compounds were analyzed by post-hoc comparison of means by Tukey’s test. Analyses were performed using the Statistica program, version 10.0 (Statsoft Inc., Tulsa, OK, USA). The level of significance was established using a two-sided *p*-value (<0.05). Pearson’s correlation coefficient was used to calculate the correlations between the dominant microbial groups with the main metabolites generated in fermentation. A principal component analysis (PCoA) based on weighted UniFrac Distances was constructed using the relative percentage peak area data of roasted coffee beans obtained on GC/MS.

## 3. Results and Discussion

### 3.1. Alpha and Beta Diversity Analysis 

A total of 800,118 and 80,740 sequences were obtained from the 16S and ITS genes, respectively. The alpha rarefaction curves from each analysis suggest that most of the microbial population was sampled (Figure S1). The microbiome involved in coffee fermentation is known to have a significant effect on the quality of the final beverage. Thus, there has been a major effort to elucidate the structure of the microbial community and the mechanisms of action behind this niche. Therefore, Shannon and Simpson indices were calculated to estimate the diversity of each sample (collected from different layers of the fermentation tank and processes conducted at different times), while richness was determined by the Chao index. The indices showed non-significant differences in microbial diversity and richness between the bottom, middle, and top layers of the fermentation tank (Table 1). However, fermentation periods longer than 24 h showed a reduction in sample richness due to a decrease in the number of subdominant species, since the Chao estimator gives more weight to microbial groups with low frequency [25]. On the other hand, bacterial diversity increased throughout fermentation while fungal diversity decreased. Diversity depends not only on richness but also on equity, i.e., homogeneity of species distribution in a community. Therefore, the increase in Shannon and Simpson values may be associated with the uniformity of the bacterial community due to the gradual increase in the number of sequences related to the Lactobacillaceae family (Table S1). 

### 3.2. Microbial Diversity and Dynamics of Fermentation

**Figure 2:**
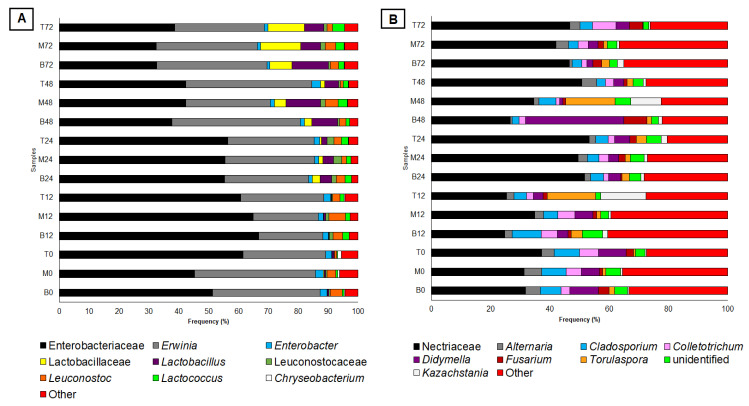
Composition of bacteria (**A**) and fungi (**B**) of samples collected in the bottom (**B**), middle (M), and top (T) layers of spontaneous coffee fermentation. Only bacteria and fungi with prevalence greater than 1% and 5% are shown, respectively. The number after each letter represents the fermentation time (e.g., B0 = bottom layer at 0 h). The complete list of minor microbial groups is reported in the Supporting Information (Table S1).

Next-generation sequencing (NGS) was used to assess the spatial distribution (i.e., bottom, middle, and top layer) of bacteria and fungi in the static-state fermentation system conducted at different times (24, 48, and 72 h). A total of 247 and 92 bacterial and fungal groups were identified, respectively (Table S1). Temporal analysis of the fermentation showed that Enterobacteriaceae family, *Erwinia*, and *Enterobacter* represented about 85% of the bacterial composition of the bottom, middle, and top layers of the fermentation tank in the first 24 h (Figure 2A). The marked presence of the Enterobacteriaceae group was also observed at the beginning of the coffee fermentation conducted in Colombia, Ecuador, Australia, and Brazil [13,23,24,26]. Vale et al. [10] and Pregolini et al. [23] showed that these bacteria are associated with coffee fruits collected from the coffee tree, fruits before pulping, and pulped fruits. 

Lactic acid bacteria (LAB) present at the beginning of fermentation were mainly represented by Leuconostocaceae family (especially *Leuconostoc* genus) and accounted for 3.94%, 2.81%, and 0.85% of the operational taxonomic unit (OTUs) identified in the bottom, middle, and top layers, respectively (Figure 2A). However, this population was not able to dominate the fermentation and remained stable throughout the fermentation process. On the other hand, a significant increase was observed in the frequency of the Lactobacillaceae family (represented mainly by *Lactobacillus*) in fermentations conducted at 48 and 72 h (Figure 2A). Moreover, a higher frequency was observed in the lower and middle layers at 24 and 48 h of fermentation compared to the top of the fermentation tank (Figure 2A). By 72 h, the relative abundance of Lactobacillaceae was approximately 20% in all three layers. This stabilization may be associated with the reduction of pH from 5.8 to 4.4 (Figure S2) since *Lactobacillus* are classified as acid-tolerant bacteria [27]. Furthermore, the acidification of the fermentation medium may also be related to the gradual reduction of enterobacteria (Figure 2A). 

Although Lactobacillaceae showed an increase after 48 h of fermentation, the total LAB population reached low proportions when compared to traditional fermentative processes performed in open tanks [13,14,27,28]. For example, the spontaneous fermentation of Colombian coffee beans performed by Junqueira et al. [24] showed that, after 48 h, LAB showed a dominance ≥90%, while in our work, the total frequency was less than 29%. LAB are historically defined as a group of microaerophilic microorganisms [29]. Therefore, the anaerobiosis conditions generated by SIAF may not be favorable for LAB growth, since other studies using this processing method also showed low populations of this bacterial group [19,20,22].

Filamentous fungi belonging to the Nectriaceae family (e.g., *Fusarium*) and the genera *Cladosporium*, *Alternaria*, *Didymella*, and *Colletotrichum* showed high populations at the beginning of the fermentative process (Figure 2B). These fungal groups have been identified as part of the microbiota of coffee leaves and fruits [10,23]. The marked presence of the Nectriaceae family and the *Fusarium* genus has also been observed during coffee fermentation from Australia and Honduras, but the role of these microorganisms has not been reported [23,26]. However, Elhalis et al. [26] suggested that the presence of these fungi is due to their ability to produce hydrolytic enzymes (e.g., cellulases and pectinases) supporting their growth during fermentation, as well as contributing to the process of mucilage removal from coffee beans. On the other hand, Vale et al. [10] associated the high presence of molds in coffee fermentation with a mechanism of fungus–rhizobacteria interaction, where bacteria are attached to fungal hyphae through fibrillar-like structures, reducing the hydrophobicity of the hyphae and promoting fungal growth in liquid medium. However, further studies are still needed to confirm any of these hypotheses. 

*Torulaspora* was the only yeast with a frequency ≥1%, detected in the bottom and middle layers at the beginning of fermentation, while other genera, such as *Pichia*, *Candida*, *Saccharomyces*, *Wickerhamomyces*, *Kazachstania*, and *Kluyveromyces*, were part of the subdominant population (Table S1). The limited yeast growth during fermentation may be associated with the low oxygen availability present in the fermentation system adopted. A study conducted by Salmon et al. [30] showed that, even under anaerobic conditions, yeasts require small amounts of dissolved oxygen to perform the biosynthesis of sterols and unsaturated fatty acids since these compounds are essential for maintaining cell viability. Some studies have also suggested that there is a positive interaction between yeast and LAB. The complex nature of these interactions is highlighted by: (i) yeast autolysis early in the fermentative process provides amino acids, polysaccharides, riboflavin, and other nutrients for LAB growth; (ii) the increase in the LAB population leads to acidification of the medium, making the environment prone to yeast development [24,31,32]. Therefore, inefficient LAB growth and slow acidification of the medium may be associated with the low yeast population throughout the fermentative process. 

### 3.3. Profile of Sugars Consumption, Organic Acids Production, and Volatile Compounds 

At the beginning of the process, a higher concentration of glucose and fructose was observed in the bottom and middle layers compared to the top of the fermentation tank (Table 2). This difference may be associated with partial precipitation of the pulp remaining in the coffee beans after the addition of water to the fermentation tank. In addition, the fructose concentration at the beginning of the fermentative process was about twice as high as glucose, which is a typical carbohydrate profile of the yellow Catuaí coffee variety [18,19]. 

Surprisingly, there was no significant consumption of sugars in the top layer in the first 48 h of fermentation. It can be speculated that microorganisms present in this layer needed more time to adapt to the anaerobic environment, as glucose only showed a significant reduction (3.06 ± 0.92 to 0.76 ± 0.01 g/L) at the end of fermentation, while fructose lasted 60 h (9.59 ± 0.72 to 5.67 ± 0.13 g/L) to be partially metabolized (Table 2). Furthermore, a marked consumption of glucose and fructose was observed in the middle layer, especially in the initial 24 h. On the other hand, there was no significant (*p* < 0.05; see Tables S2 and S3) reduction of fructose in the bottom layer during the whole fermentative process, while glucose was metabolized only in the first 24 h, followed by a stabilization in concentration until the end of fermentation. After 72 h, the residual concentration of glucose and fructose in the fermentation tank was 6.27 and 35.10 g/L, respectively. The presence of residual sugars is commonly reported in spontaneous coffee fermentations conducted in different countries (Brazil, Australia, Colombia, and Ecuador) and are usually associated with short fermentation cycles, i.e., between 24 and 36 h [24,26,32,33,34]. However, the results showed that only increasing the fermentation time does not guarantee that the indigenous microbiota can consume all the sugars in the mucilage, especially fructose. Thus, an alternative to optimizing fructose consumption is the use of starter cultures that exhibit a fructophilic phenotype, as recently suggested by Junqueira et al. [35]. 

**Figure 3:**
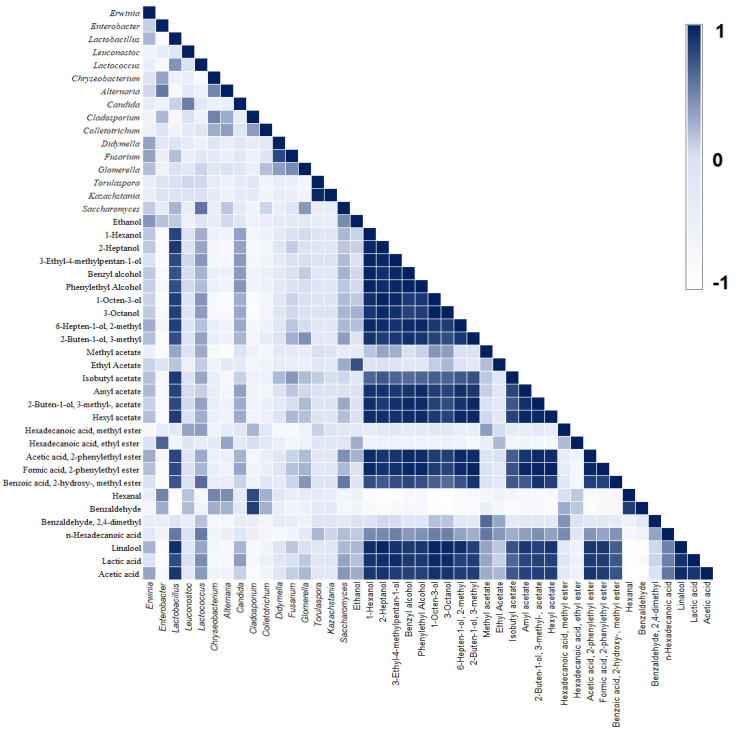
Pearson correlation matrix of the main microbial groups and volatile compounds identified.

Lactic and acetic acids were the main organic acids produced during fermentation. Lactic acid showed a significant increase (*p* < 0.05), reaching a final concentration of 5.06 ± 0.02, 4.70 ± 0.02, and 2.18 ± 0.01 g/L in the bottom, middle, and top layers, respectively (Table 2); it showed a positive correlation (≥0.45) with microorganisms belonging to the genera *Lactobacillus*, *Lactococcus*, *Candida*, and *Saccharomyces* (Figure 3). However, most of the lactic acid was probably produced by *Lactobacillus*, as its population gradually increased from the bottom to the top layer during fermentation. In addition, the lower concentration of lactic acid observed at the top of the fermentation tank may be associated with slower glucose metabolism, as discussed earlier. The production of this organic acid is of great importance for fermentation because it helps in the process of acidification of the medium without interfering with the quality of the final beverage, besides inhibiting the growth of undesirable microorganisms [36].

Acetic acid was detected after 18 h of fermentation and its production gradually increased during the fermentative process (Table 2). However, production was not related to the presence of AAB since this microbial group showed less than 1% of read sequences. Furthermore, AAB is known to be strictly aerobic, being able to oxidize alcohols, aldehydes, sugars, or sugar alcohols only in the presence of oxygen [37,38]. Therefore, it is speculated that acetic acid production is associated with the ability of some *Lactobacillus* strains to degrade lactic acid into acetic acid under anaerobic conditions and in acidic environments [39]. Although the exact function of this pathway is unclear, some results suggest that it may be associated with the maintenance of cell viability [39]. Furthermore, a positive correlation (0.90) of bacteria belonging to the genus *Lactobacillus* with acetic acid was observed (Figure 3). 

**Figure 4:**
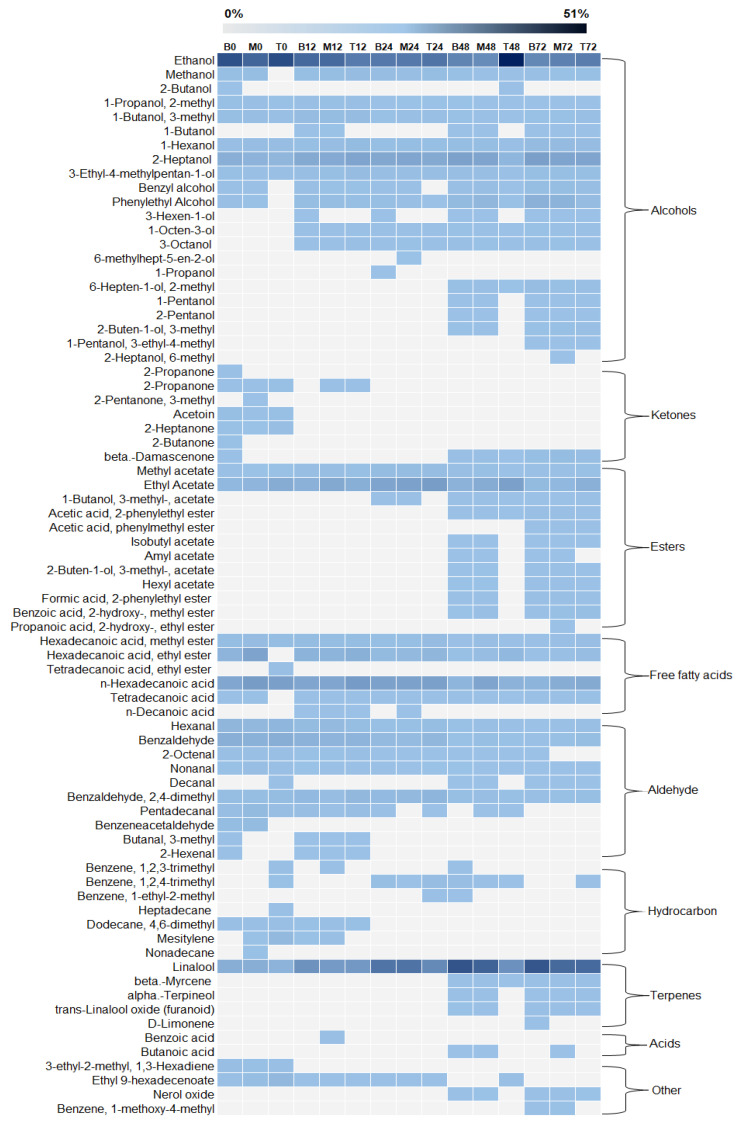
Heatmap of the volatile compounds identified in the bottom (B), middle (M), and top (T) layers. The number after each letter represents the fermentation time (e.g., B0 = bottom layer at 0 h).

**Figure 5:**
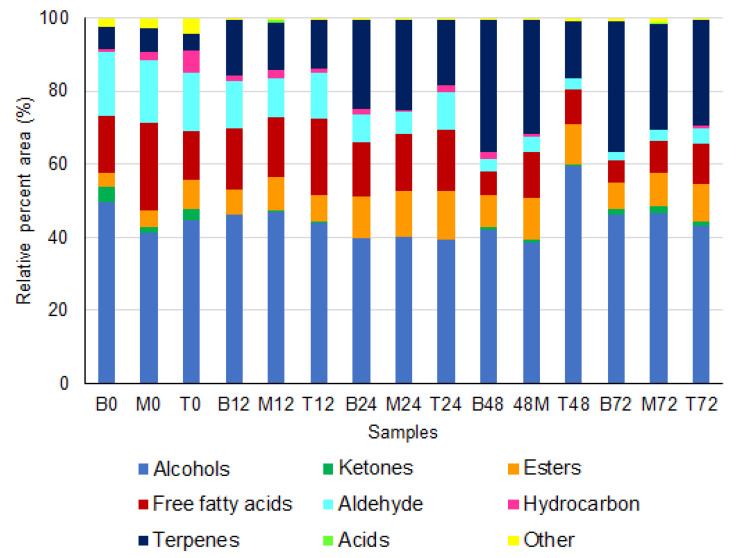
Chemical groups identified in the bottom (B), middle (M), and top (T) layers in the fermentation liquid fraction. The number after each letter represents the fermentation time (e.g., B0 = bottom layer at 0 h).

A total of 76 volatile compounds were identified by HS-SPME/GC in the fermentation liquid fraction. These compounds were grouped into nine chemical groups: alcohols (22), ketones (7), esters (13), free fatty acids (6), aldehydes (10), hydrocarbons (7), terpenes (5), acids (2), and others (4) are shown in (Figure 4 and Table S6). Alcohols were the chemical group with the highest relative percentage of peak areas (38.5–59.5%) and, although the intensity of this group varied throughout the fermentation, only the top layer at 48 h showed a significant difference from the other samples (Figure 5). This difference is mainly associated with a peak in ethanol production, which can be attributed to a discrete increase in the frequency of *Saccharomyces* yeast at the top layer at 48 h. Figure 3 shows the positive correlation between *Saccharomyces* and ethanol. The fermentation time was an important variable in the production of molecules belonging to alcohols since, at the beginning of the fermentation process, only 10 volatile compounds were identified and, after 72 h, 22 molecules were produced (e.g., 1-Octen-3-ol; 3-Octanol; 6-Hepten-1-ol; 2-methyl, 2-Buten-1-ol, and 3-methyl) as shown in Figure 4. Moreover, long fermentation periods also allowed the accumulation of several alcohols (e.g., 1-Hexanol, 2-Heptanol, Benzyl alcohol, and Phenylethyl alcohol) in the liquid fraction of the fermentation and, at the end of the process, the rate of these compounds was also higher in the bottom and middle layers compared to the top of the fermentation tank. However, the relationship between alcohols and coffee quality has not yet been well established, but this chemical group is known for its high sensory threshold. Thus, it is speculated that to modulate the quality of the final beverage, a high concentration of these compounds and an intense diffusion process into coffee beans are required [6,40].

Esters also showed a relatively higher abundance when compared to other coffee fermentation studies [10,23,24,32]. At the beginning of fermentation, this chemical group was mainly represented by methyl acetate and ethyl acetate. Fermentation time was also an important variable for ester production, as, after 48 h, 10 more compounds were produced (e.g., isobutyl acetate, amyl acetate, 2-buten-1-ol, 3-methyl-acetate, hexyl acetate, among others) that remained until the end of fermentation (Figure 4 and Table S6). Interestingly, the production of these molecules also showed a high correlation with *Lactobacillus* and alcohols, which was expected, since the generation of esters by *Lactobacillus* occurs through an esterification reaction between fatty acids and an alcohol molecule [36]. The accumulation of esters is highly desired, as they can impart floral, fruity, and buttery perceptions in the coffee beverage, even if they are in low concentrations [6].

The terpenes were mainly represented by linalool, which showed a significant increase (*p* < 0.05) during the fermentation process. However, like the alcohols and esters, new terpenoids (beta-Myrcene, D-Limonene, trans-Linalool oxide (furanoid), and alpha-Terpineol) were produced in fermentations conducted for longer periods (48 and 72 h). Generally, these compounds originate from the activity of β-glycosidases that release monoterpenes from glycosidically bound precursors [41,42]. In addition, it is speculated that some yeast species may also produce these molecules via the mevalonic acid pathway [27,43]. However, the formation of these compounds during coffee fermentation and the impact on the coffee quality is still unclear. On the other hand, no accumulation or production of new compounds belonging to aldehydes and ketones, which are frequently identified in the liquid fraction of spontaneous coffee fermentations, was observed. However, a gradual reduction of some aldehydes (e.g., hexanal, benzaldehyde) is noted throughout the fermentation, while ketones were only detected in the initial 12 (Figure 4).

We speculate that this reduction may be associated with a diffusion process into the coffee beans or that these molecules were precursors for the formation of other volatile compounds [11,19]. Finally, the results showed that, in general, the consumption of sugars and the production of organic acids and volatile compounds showed significant differences among the bottom, middle, and top layers throughout the fermentation, suggesting that although the bacterial and fungal diversity did not show great variations, the metabolism of these microorganisms can change drastically among the layers. Furthermore, the use of a closed fermentation tank can decrease the loss of specific volatile compounds to the environment and increase the diffusion rate of these molecules into the coffee beans; however, this process can be optimized if there is an agitation/revolving process, as recently demonstrated in other works [15,16]. 

### 3.4. Volatile Composition of Green and Roasted Coffee Beans 

#### 3.4.1. Green Coffee Beans 

A total of 29 compounds were identified in the green coffee beans. These volatiles were grouped according to chemical class (Table 3). Ethanol and 1-Hexanol were the main compounds detected. The ethanol content in coffee beans fermented for 72 h was about six times higher than in the beans fermented for 24 h. This accumulation of ethanol may be associated with the induction of a fermentative metabolism due to the hypoxic conditions imposed by the fermentative process [44]. Interestingly, some alcohols such as 2-Propanol, 1-methoxy; 2-Hexanol, 5-methyl, 3-Furanmethanol, were only identified in coffee beans fermented for 24 h, while 1-Propanol, 2 methyl was unique to the beans fermented for 72 h (Table 3). The increase in fermentation time resulted in the gradual decrease of some compounds belonging to the aldehydes, for instance: hexanal, nonanal, butanal, 3-methyl, and heptanal. A recent work by Salem et al. [11] evaluated the diffusion process of ester, alcohol, and aldehyde into coffee beans and showed that butanal was the compound that showed the fastest decrease among the molecules evaluated. The authors suggest that this reduction is due to a degradation mechanism of this compound inside the coffee beans. 

Among the esters, only ethyl acetate and pentatonic acid ethyl ester were detected in coffee beans fermented for 72 h. Although ethyl acetate was the main ester detected in the liquid fraction of the fermentation, the presence of this compound was not observed in the green beans fermented for 24 and 48 h. Thus, it is speculated that the diffusion process of ethyl acetate may be relatively slow. Furthermore, the results indicate that other compounds (e.g., 1-Hexanol, 1-pentanol, 1-Butanol, 3-methyl, 1-Propanol, 2-methyl, and 2-pentanone, 3-methyl) detected in the green coffee beans may also come from microbial metabolism.

#### 3.4.2. Roasted Beans 

A total of 70 compounds were identified among the roasted coffee beans. The most abundant chemical groups (i.e., largest relative peak area) were furans, alcohols, ketones, pyrazines, and acids. In general, there was not much variation in the number of volatile compounds identified among the coffee beans fermented by 24, 48, and 72 h. However, a significant increase in the content of several compounds (e.g., 2-furancarboxaldehyde 5-methyl; furfural; ethanol; 2-propanone, 1-hydroxy; 2,3-Pentanedione; 4-hydroxy-3-methylacetophenone; 2-furancarboxylic acid, methyl ester; butanal, 3-methyl; acetic acid among others) is noted in the coffee beans fermented for long periods (48 and 72 h), as shown in (Table 4). These compounds are formed by numerous biochemical reactions (e.g., Maillard reactions, pyrolysis, and Strecker degradation) that occur during the roasting process. For example, furans are heterocyclic compounds that exhibit high volatility and are generated during the Maillard reaction, with sucrose, glucose, and linoleic acid being their potential precursors [21,45].

The alcohols were the second most abundant chemical group. This high content is mainly due to the accumulation of 3-furanomethanol, and its production can be partially attributed to the Cannizzaro reaction, in which furfural acts as a reactant [46,47]. However, the impact of this chemical compound on the final beverage is not yet known. On the other hand, ketones (e.g., 2,3-Pentanedione, acetoin, 2-propanone) and pyrazines (e.g., pyrazine, 2,3-dimethyl, pyrazine, 2-ethyl-6-methyl), detected in roasted beans, can impart a buttery, creamy, sweet, nutty, and fruity flavor in the final coffee beverage [6].

Acetic acid was the main organic acid detected in roasted coffee beans. However, the impact of acetic acid on coffee quality is still unclear [18,48]. For example, Chindapan et al. [49] speculated that acetic acid and formic acid may contribute to coffee acidity, as well as impart a fruit-like flavor when they are at low concentrations, but an unpleasant taste at higher concentrations. However, the authors did not determine this threshold for roasted coffee beans. On the other hand, Liu et al. [50] showed that pre-treating green coffee beans with acetic acid were able to significantly increase the quality of the final product. Interestingly, roasted beans fermented for 48 h showed a higher rate of ester and aldehydes, as shown in (Table 4). Like ketones and pyrazines, these chemical groups can also attribute a fruity, floral, and buttery aroma; however, there should be a balance between the compounds present in coffee beans, since the overall flavor of the coffee depends not only on the concentration but also on the detection threshold of each compound and its interactions with other volatile and non-volatile molecules [6]. 

### 3.5. Sensory Analysis

**Figure 6:**
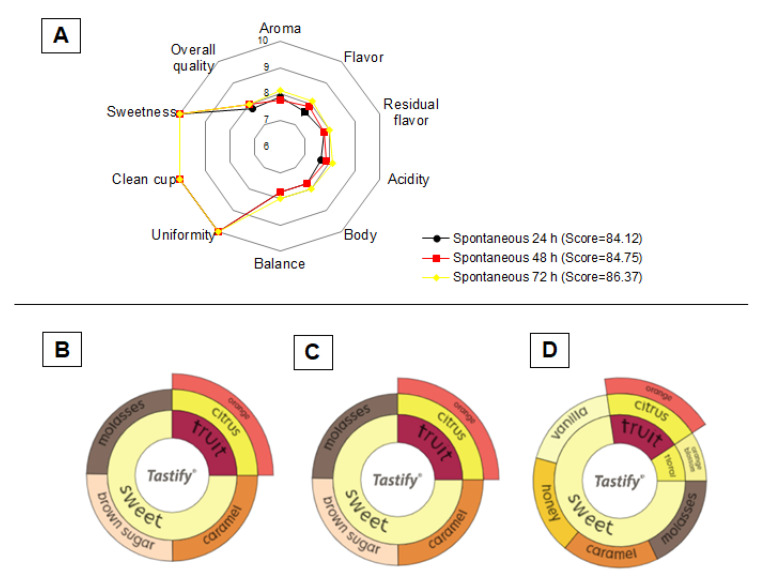
Sensorial evaluation of beverages produced with roasted coffee beans. Final score of the processed coffees for different times (**A**) and description of the sensorial attributes of the fermented coffees for 24 h (**B**), 48 h (**C**), and 72 h (**D**).

**Figure 7:**
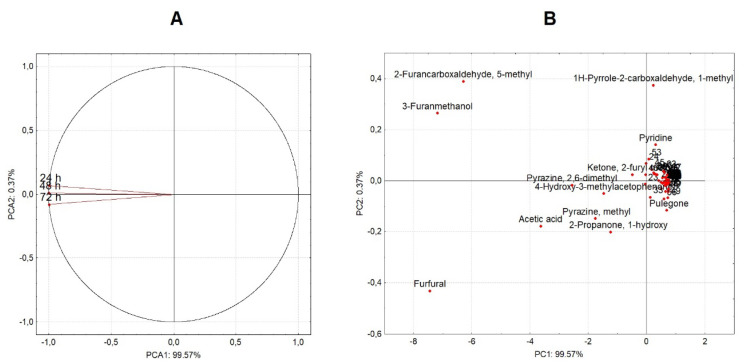
A principal component analysis based on weighted UniFrac Distances. (**A**) Analysis of volatile compounds detected in roasted coffee beans processed for 24, 48, and 72 h and (**B**) dispersion of these compounds in the three treatments.

Coffee beverages produced from coffee beans fermented for 24, 48, and 72 h were evaluated by certified Q-Graders according to the SCA methodology All the coffee beverages scored ≥ 84 and were classified as specialty coffees (Figure 6). In general, the increase in fermentation time impacted the perception of aroma, flavor, acidity, body, overall quality, and balance attributes, while sweetness, clean cup, and uniformity showed no difference between treatments. Thus, coffee beans fermented for 48 and 72 h resulted in beverages with greater complexity and intensity compared for 24 h (Figure 6). Intensity is related to acidity, and it is noted that this sensory attribute is highly influenced by processing time. In addition, the presence of acetic acid and formic acid in roasted beans may be associated with the fruity perception detected by the Q-Graders in all treatments [49,51]. The body and aftertaste of the beverages produced from the beans processed for 24 and 48 h were described by the Q-Grader as “medium pleasant” and “medium creamy,” while the coffee beans fermented for 72 h were “velvety/dense” and “long silky” (data not shown). To explain the differences observed in the sensory profiles of coffees processed at different times, PCA analysis was constructed based on the relative percentage of volatile compounds identified in the roasted coffee beans (Table 4 and Figure 7). In general, most of the volatiles are part of a single group (Figure 7B). Although the beverage flavor is shaped by a series of interactions between all constituents of the coffee beans, it can be speculated that the differences in the percentage of some compounds (e.g., acetic acid, furfural, pyrazine, methyl, 2-Propanone, 1-hydroxy among others) may be related to the different sensory profiles obtained in this study (Figure 6 and Figure 7). Finally, these results show a positive influence of time on the quality of the final coffee beverage.

## 4. Conclusions

The SIAF method showed high prevalence of Enterobacteriaceae and filamentous fungi and low prevalence of LAB and yeast. The anaerobic conditions may be the main factor that influenced the growth of LAB and yeast, but further studies should be investigated to prove this hypothesis. Significant variations were observed in sugar consumption and metabolite formation between the different layers of the sampled tank. Agitation of the fermentation tank can be used as a potential way to homogenize microbial metabolism during SIAF, as observed in several other industrial processes. However, further studies are needed to assess the influence of a dedicated turning device for a coffee fermentation tank and coffee beverage quality. Finally, variations in fermentation time showed be a potential tool for delivering coffee beverages with novel and desirable flavor profiles.

## Figures and Tables

**Table 1:** Microbial community richness and diversity in different layers of a static coffee beans fermentation tank conducted at different times (24, 48, and 72 h).

Sample	Bacteria Indices			Fungi Indices		
	Chao	Shannon	Simpson	Chao	Shannon	Simpson
B0	1686.7	1.56	0.68	149.4	2.79	0.87
M0	1391	1.65	0.69	143.9	2.73	0.87
T0	1526.9	1.52	0.64	179.5	2.59	0.83
B12	1740.6	1.44	0.63	179.5	2.98	0.90
M12	1114.1	1.40	0.61	110.2	2.67	0.85
T12	1637.9	1.53	0.66	108.6	2.64	0.87
B24	1424.5	1.56	0.67	137.4	2.19	0.71
M24	1358.3	1.53	0.67	117.8	2.26	0.73
T24	1774.6	1.59	0.68	111.5	2.19	0.70
B48	1628	1.61	0.69	102.4	2.24	0.80
M48	1444.5	1.89	0.77	83.8	2.40	0.83
T48	1358.3	1.62	0.70	74.3	2.16	0.72
B72	1142.7	1.84	0.76	85.2	2.00	0.75
M72	1216	1.94	0.78	88.9	2.52	0.80
T72	1293.3	1.93	0.78	89	2.19	0.75

B = sample taken at the bottom of the fermentation tank; M = sample taken at the middle of the fermentation tank; T = sample taken at the top of the fermentation tank. 0, 12, 24, 48, 72 h of fermentation time.

**Table 2:** Sugar consumption and organic acids production (g l^−1^) during spontaneous fermentation.

Time (h)	Glucose			Fructose			Lactic Acid			Acetic Acid		
	Bottom	Middle	Top	Bottom	Middle	Top	Bottom	Middle	Top	Bottom	Middle	Top
0	9.12 ± 0.42	8.52 ± 1.05	3.06 ± 0.92	20.65 ± 0.90	16.23 ± 0.77	9.59 ± 0.72	ND	ND	ND	ND	ND	ND
6	8.03 ± 1.16	5.43 ± 1.31	4.4 ±0.82	20.37 ± 0.92	18.14 ± 0.98	13.41 ± 0.25	0.63 ± 0.09	0.41 ± 0.05	0.28 ± 0.08	ND	ND	ND
12	7.66 ± 0.87	5.57 ± 0.87	3.93 ± 0.77	20.61 ± 1.88	15.59 ± 0.41	10.92 ± 1.68	0.91 ± 0.19	0.78 ± 0.23	0.43 ± 0.02	ND	ND	ND
18	6.88 ± 0.23	4.49 ± 1.33	3.53 ± 0.27	19.25 ± 0.59	14.46 ± 1.91	10.98 ± 0.35	2.06 ± 0.38	1.60 ± 0.44	1.10 ± 0.07	0.25 ± 0.03	0.24 ± 0.06	0.14 ± 0.01
24	4.74 ± 0.94	3.63 ± 0.32	3.06 ± 0.61	16.36 ± 0.57	12.37 ± 0.70	8.52 ± 0.14	2.11 ± 0.23	1.66 ± 0.05	1.00 ± 0.02	0.38 ± 0.11	0.36 ± 0.03	0.19 ± 0.00
30	4.56 ± 0.25	2.77 ± 0.32	2.43 ± 0.03	16.10 ± 0.94	10.63 ± 1.04	8.98 ± 0.55	2.36 ± 0.22	2.15 ± 0.02	1.36 ± 0.16	0.54 ± 0.11	0.48 ± 0.06	0.24 ± 0.05
36	5.07 ± 0.70	2.58 ± 0.30	2.29 ± 0.28	17.96 ± 2.00	11.28 ± 1.12	8.19 ± 1.30	2.77 ± 0.28	2.26 ± 0.23	1.39 ± 0.25	0.59 ± 0.06	0.51 ± 0.03	0.26 ± 0.05
48	4.31 ± 0.51	2.04 ± 0.23	1.91 ± 0.14	16.23 ± 1.55	10.62 ± 0.66	9.23 ± 0.39	3.18 ± 0.27	2.83 ± 0.22	2.13 ± 013	0.67 ± 0.00	0.63 ± 0.06	0.50 ± 0.02
60	4.36 ± 0.06	1.23 ± 0.07	1.06 ± 0.04	17.76 ± 0.49	9.56 ± 0.20	5.67 ± 0.13	4.31 ± 0.23	3.24 ± 0.06	2.03 ± 0.02	0.88 ± 0.06	0.71 ± 0.01	0.40 ± 0.04
72	4.47 ± 0.04	1.04 ± 0.12	0.76 ± 0.01	18.67 ± 0.80	11.09 ± 0.46	5.34 ± 0.07	5.06 ± 0.02	4.70 ± 0.02	2.18 ± 0.01	1.00 ± 0.11	0.94 ± 0.00	0.40 ± 0.04

ND = not detected. Statistical analysis is reported in the Supplementary Materials (Tables S2–S5).

**Table 3:** Volatile compounds (area × 10^5^) identified in fermented green coffee beans.

Compounds	Green Beans		
	24 h	48 h	72 h
Alcohols			
Ethanol	0.73 ± 0.03 ^A^	2.30 ± 0.27 ^B^	6.69 ± 0.34 ^C^
2-Propanol, 1-methoxy	0.10 ± 0.01	ND	ND
1-Butanol, 3-methyl	0.20 ± 0.02 ^A^	0.34 ± 0.01 ^B^	0.21 ± 0.02 ^A^
1-Pentanol	0.28 ± 0.03 ^AB^	0.26 ± 0.02 ^A^	0.35 ± 0.02 ^B^
2-Hexanol, 5-methyl	0.30 ± 0.28	ND	ND
2,3-Butanediol	0.24 ± 0.01 ^AB^	0.20 ± 0.01 ^A^	0.25 ± 0.01 ^B^
3-Furanmethanol	0.55 ± 0.02	ND	ND
1-Hexanol	1.30 ± 0.02 ^A^	1.15 ± 0.09 ^B^	0.81 ± 0.02 ^C^
1-Propanol, 2 methyl	ND	ND	0.03 ± 0.00
Aldehydes			
Butanal, 3-methyl	0.19 ± 0.03 ^A^	0.16 ± 0.01 ^A^	0.15 ± 0.01 ^A^
Butanal, 2-methyl	0.11 ± 0.01 ^A^	0.10 ± 0.01 ^A^	0.07 ± 0.00 ^B^
Pentanal	0.31 ± 0.03 ^A^	0.27 ± 0.03 ^AB^	0.21 ± 0.03 ^A^
Hexanal	1.51 ± 0.16 ^A^	1.19 ± 0.28 ^AB^	0.92 ± 0.06 ^B^
Heptanal	0.16 ± 0.07 ^A^	0.12 ± 0.02 ^A^	0.09 ± 0.00 ^A^
Nonanal	0.53 ± 0.02 ^A^	0.46 ± 0.10 ^AB^	0.33 ± 0.05 ^B^
Decanal	0.16 ± 0.01 ^A^	0.15 ± 0.03 ^A^	ND
Furans			
Furan, 2-methyl	0.12 ± 0.02 ^A^	0.10 ± 0.00 ^A^	0.22 ± 0.03 ^B^
Furan, 2-pentyl	0.16 ± 0.01 ^A^	0.22 ± 0.01 ^A^	0.19 ± 0.04 ^A^
Esters			
Ethyl Acetate	ND	ND	0.21 ± 0.01
Pentanoic acid, ethyl ester	ND	ND	0.17 ± 0.02
Acids			
Butanoic acid, 3-methyl	0.21 ± 0.02 ^A^	0.16 ± 0.01 ^A^	0.18 ± 0.03 ^A^
Acetic acid	0.20 ± 0.00 ^A^	0.62 ± 0.24 ^B^	0.47 ± 0.06 ^AB^
Ketones			
2-Propanone, 1-hydroxy	0.10 ± 0.00	ND	ND
5-Hepten-2-one, 6-methyl	0.08 ± 0.00 ^A^	0.04 ± 0.00 ^B^	0.11 ± 0.00 ^C^
2-Propanone	ND	0.85 ± 0.11	ND
2-Pentanone, 3-methyl	ND	0.04 ± 0.00 ^A^	0.03 ± 0.01 ^A^
Pyrazines			
2-Isobutyl-3-methoxypyrazine	0.93 ± 0.01 ^A^	1.03 ± 0.12 ^A^	0.72 ± 0.05 ^B^
Terpenes			
D-Limonene	ND	0.13 ± 0.02	ND
Hydrocarbon			
Nonane, 3-methyl-5-propyl	0.12 ± 0.01 ^A^	0.11 ± 0.00 ^A^	0.07 ± 0.00 ^B^

ND = not detected. Means of triplicate in each row bearing the same letters (A–C) are not significantly different (*p* > 0.05) from one another using Tukey’s test (mean standard variation).

**Table 4:** Volatile compounds (area × 10^5^) identified in roasted beans.

Compounds	Roasted Beans		
	24 h	48 h	72 h
Furans			
Furan, 2-methyl	0.37 ± 0.06 ^A^	1.46 ± 0.24 ^B^	1.00 ± 0.11 ^C^
Furan, 2,5-dimethyl	0.06 ± 0.01 ^A^	0.12 ± 0.01 ^B^	0.12 ± 0.01 ^B^
2-Vinylfuran	0.23 ± 0.07 ^A^	0.26 ± 0.02 ^A^	0.22 ± 0.04 ^A^
3(2H)-Furanone, dihydro-2-methyl	1.87 ± 0.03 ^A^	2.72 ± 0.12 ^B^	2.63 ± 0.25 ^B^
Furfuryl formate	2.44 ± 0.12 ^A^	2.90 ± 0.02 ^B^	2.69 ± 0.12 ^AB^
2,5-Dimethylfuran-3,4(2H,5H)-dione	2.39 ± 0.16 ^A^	3.24 ± 0.21 ^B^	3.93 ± 0.08 ^C^
5-Ethylfurfural	ND	ND	0.30 ± 0.01
Furfural	77.06 ± 2.54 ^A^	101.21 ± 1.78 ^B^	110.10 ± 2.09 ^C^
2-Furancarboxaldehyde, 5-methyl	75.75 ± 2.74 ^A^	87.10 ± 3.56 ^B^	82.83 ± 3.49 ^AB^
Alcohols			
Ethanol	ND	0.22 ± 0.02 ^A^	1.12 ± 0.05 ^B^
3-Furanmethanol	84.08 ± 3.09 ^A^	96.17 ± 3.65 ^B^	96.76 ± 1.58 ^B^
Propanol, 2 methyl	ND	0.63 ± 0.05	ND
Ketones			
2-Propanone, 1-hydroxy	17.44 ± 0.53 ^A^	25.24 ± 2.32 ^B^	28.24 ± 2.26 ^B^
2,3-Pentanedione	2.08 ± 0.12 ^A^	2.99 ± 0.16 ^B^	3.03 ± 0.23 ^B^
Acetoin	0.67 ± 0.03 ^A^	1.31 ± 0.05 ^B^	1.28 ± 0.04 ^B^
2-Propanone	1.64 ± 0.21 ^A^	1.73 ± 0.13 ^A^	1.64 ± 0.05 ^A^
1-Hydroxy-2-butanone	1.02 ± 0.04 ^A^	1.47 ± 0.12 ^B^	1.59 ± 0.12 ^B^
3-Pentanone, 2-methyl	0.69 ± 0.02	ND	ND
Ketone, 2-furyl methyl	12.77 ± 0.49 ^A^	15.92 ± 0.43 ^B^	15.33 ± 0.32 ^B^
2,5-Hexanedione	0.43 ± 0.02 ^A^	0.55 ± 0.01 ^A^	0.46 ± 0.09 ^A^
2-Butanone, 1-hydroxy-, acetate	1.43 ± 0.15 ^A^	1.59 ± 0.05 ^A^	2.26 ± 1.34 ^A^
1-Propanone, 1-(2-furanyl)	0.55 ± 0.06 ^A^	0.79 ± 0.03 ^A^	0.68 ± 0.04 ^A^
3-Ethyl-2-hydroxy-2-cyclopenten-1-one	0.90 ± 0.06 ^A^	1.07 ± 0.03 ^A^	0.97 ± 0.03 ^A^
2-Acetyl-3-methylpyrazine	8.47 ± 0.43 ^A^	9.15 ± 0.60 ^A^	10.83 ± 0.32 ^A^
2-Propanone, 1-hydroxy-, acetate	8.68 ± 0.35 ^A^	10.03 ± 0.33 ^A^	8.82 ± 0.30 ^A^
2-Acetyl-5-methylfuran	4.38 ± 0.54 ^A^	5.16 ± 0.39 ^A^	4.77 ± 0.07 ^A^
Resorcinol, 2-acetyl	3.42 ± 0.56 ^A^	4.95 ± 0.50 ^A^	4.36 ± 0.04 ^A^
4-Hydroxy-3-methylacetophenone	21.92 ± 4.30 ^A^	27.58 ± 2.84 ^A^	29.02 ± 0.20 ^A^
Pyrazines			
1,3-Diazine	1.71 ± 0.13 ^A^	2.07 ± 0.04 ^AB^	2.27 ± 0.21 ^B^
Pyrazine, methyl	24.12 ± 0.28 ^A^	29.88 ± 0.74 ^B^	34.60 ± 1.45 ^C^
Pyrazine, 2,6-dimethyl	34.55 ± 1.30 ^A^	38.35 ± 0.14 ^A^	43.27 ± 2.48 ^B^
Pyrazine, ethyl	2.91 ± 0.19 ^A^	3.31 ± 0.08 ^AB^	3.75 ± 0.33 ^B^
Pyrazine, 2,3-dimethyl	0.99 ± 0.08 ^A^	1.13 ± 0.05 ^AB^	1.26 ± 0.10 ^B^
Pyrazine, ethenyl	0.84 ± 0.13 ^A^	1.10 ± 0.11 ^B^	1.20 ± 0.00 ^B^
Pyrazine, 2-ethyl-6-methyl	5.67 ± 0.27 ^AB^	4.54 ± 0.18 ^A^	6.02 ± 0.75 ^B^
Pyrazine, trimethyl	6.27 ± 0.17 ^A^	6.74 ± 0.15 ^A^	9.59 ± 0.52 ^B^
Pyrazine, 3-ethyl-2,5-dimethyl	6.35 ± 0.28 ^A^	5.40 ± 0.16 ^B^	6.70 ± 0.31 ^A^
1-(6-Methyl-2-pyrazinyl)-1-ethanone	1.81 ± 0.17 ^A^	1.77 ± 0.07 ^A^	2.26 ± 0.03 ^B^
Pyrazine, 2-methyl-5-(1-propenyl)-, (E)	0.99 ± 0.17 ^A^	1.16 ± 0.11 ^A^	1.14 ± 0.04 ^A^
Pyrazine, 2-methyl-6-propenyl	ND	ND	1.23 ± 0.02
Pyrazine, 2,3-diethyl-5-methyl	ND	ND	0.23 ± 0.00
Acids			
Butanoic acid, 3-methyl	1.06 ± 0.17 ^A^	1.95 ± 0.09 ^B^	1.96 ± 0.31 ^B^
Butanoic acid, 2-methyl	ND	ND	0.18 ± 0.04
Acetic acid	42.44 ± 4.45 ^A^	53.49 ± 3.10 ^B^	58.60 ± 0.73 ^B^
Formic acid	0.62 ± 0.13 ^A^	1.14 ± 0.05 ^B^	1.24 ± 0.08 ^B^
Propanoic acid	0.92 ± 0.07 ^A^	1.24 ± 0.05 ^B^	1.22 ± 0.08 ^B^
2-Butenoic acid, 3-methyl	0.58 ± 0.02 ^A^	1.76 ± 0.07 ^B^	2.05 ± 0.09 ^C^
Pyrroles			
1H-Pyrrole-2-carboxaldehyde	6.35 ± 0.58 ^A^	7.67 ± 0.45 ^B^	6.65 ± 0.26 ^AB^
Ketone, methyl pyrrol-2-yl	8.34 ± 0.61 ^A^	9.91 ± 0.71 ^B^	9.71 ± 0.17 ^AB^
Ethanone, 1-(1-methyl-1H-pyrrol-2-yl)	1.51 ± 0.13 ^A^	1.57 ± 0.03 ^A^	1.43 ± 0.27 ^A^
1H-Pyrrole-2-carboxaldehyde, 1-methyl	8.90 ± 0.07 ^A^	9.09 ± 0.71 ^A^	ND
1H-Pyrrole, 1-(2-furanylmethyl)	1.80 ± 0.13 ^A^	1.86 ± 0.06 ^A^	1.76 ± 0.04 ^A^
Pyrrole	ND	ND	1.93 ± 0.14
Esters			
Methyl acetate	0.31 ± 0.07 ^A^	0.29 ± 0.01 ^A^	0.27 ± 0.02 ^A^
Propanoic acid, 2-oxo-, methyl ester	0.20 ± 0.03 ^A^	0.20 ± 0.03 ^A^	0.21 ± 0.03 ^A^
Acetic acid, 4-methylphenyl ester	0.38 ± 0.06 ^A^	1.90 ± 0.08 ^B^	ND
Furfuryl acetate	7.97 ± 0.26 ^A^	8.77 ± 0.12 ^B^	7.38 ± 0.27 ^C^
2-Furancarboxylic acid, methyl ester	1.56 ± 0.17 ^A^	2.04 ± 0.20 ^B^	2.04 ± 0.04 ^B^
Pyrans			
4H-Pyran-4-one, 3-hydroxy-2-methyl	5.20 ± 0.86 ^A^	6.84 ± 0.58 ^B^	5.77 ± 0.17 ^AB^
4H-Pyran-4-one, 2,3-dihydro-3,5-dihydroxy-6-methyl	1.05 ± 0.08 ^A^	1.88 ± 0.15 ^B^	3.46 ± 0.18 ^C^
Pyridines			
Pyridine	4.91 ± 0.34 ^A^	8.63 ± 0.45 ^B^	2.37 ± 0.03 ^C^
Pyridine, 2-methyl	0.17 ± 0.07 ^AB^	0.17 ± 0.01 ^A^	0.28 ± 0.00 ^B^
Aldehydes			
Propanal, 2-methyl	0.46 ± 0.03 ^A^	0.63 ± 0.05 ^B^	0.75 ± 0.05 ^B^
Butanal, 3-methyl	0.40 ± 0.04 ^A^	0.64 ± 0.01 ^B^	0.72 ± 0.03 ^B^
Benzeneacetaldehyde	1.88 ± 0.06 ^A^	2.34 ± 0.07 ^B^	2.18 ± 0.04 ^C^
Other			
Cyclopent-4-ene-1,3-dione	1.30 ± 0.09 ^A^	1.74 ± 0.07 ^B^	1.72 ± 0.22 ^B^
1,2-Cyclopentanedione, 3-methyl	2.93 ± 0.93 ^A^	2.23 ± 0.10 ^A^	1.95 ± 0.08 ^A^
4-Methylthiazole	ND	ND	0.15 ± 0.03
2-Cyclopenten-1-one, 3-ethyl-2-hydroxy	ND	ND	0.90 ± 0.04
Terpenes			
Pulegone	ND	ND	3.37 ± 0.09

ND = not detected. Means of triplicate in each row bearing the same letters (A–C) are not significantly different (*p* > 0.05) from one another using Tukey’s test (mean standard variation).

## Data Availability

Illumina reads were deposited on NCBI’s GenBank under the BioProject ID PRJNA913882 and the BioSamples ID SAMN32310725.

## Supplementary Materials

The following supporting information can be downloaded at: https://www.mdpi.com/article/10.3390/foods12010037/s1, Figure S1: Alpha rarefaction curves of the OTUs (operational taxonomic units) observed from the fermentation liquid fraction samples. (A) Bacterial analysis, (B) Fungal analysis; Figure S2: Temperature and pH analysis of the bottom (B), middle (M), and top (T) layers during spontaneous fermentation. The number after each letter represents the fermentation time (e.g., B0 = bottom layer at 0 h); Table S1: Relative abundance (%) of bacteria and fungi identified during fermentation of Brazilian coffee beans; Table S2: Statistical analysis of glucose consumption; Table S3: Statistical analysis of fructose consumption; Table S4: Statistical analysis of lactic acid production; Table S5: Statistical analysis of acetic acid production; Table S6: Statistical analysis of volatile compounds detected in the liquid fraction from fermentation of Brazilian coffee beans.
