# Supplementary material for: Understanding the Effects of Self-Induced Anaerobic Fermentation on Coffee Beans Quality: Microbiological, Metabolic, and Sensory Studies
Source: Foods. 2022 Dec 22;12(1):37. doi: 10.3390/foods12010037 (PMC9818356; doi:10.3390/foods12010037)
Supplement: Supplementary file 1 [file foods-12-00037-s001.zip › foods-2087229-supplementary.pdf]

Understanding the effects of self-induced anaerobic fermentation on coffee beans quality: microbiological, metabolic, and sensory studies

Alexander da Silva Vale[1], Gabriel Balla[1], Luiz Roberto Saldanha Rodrigues[1], Dão Pedro de Carvalho Neto[2], Carlos Ricardo Soccol[1], Gilberto Vinícius de Melo Pereira[1,*]

Supplementary Material

Supplementary Material

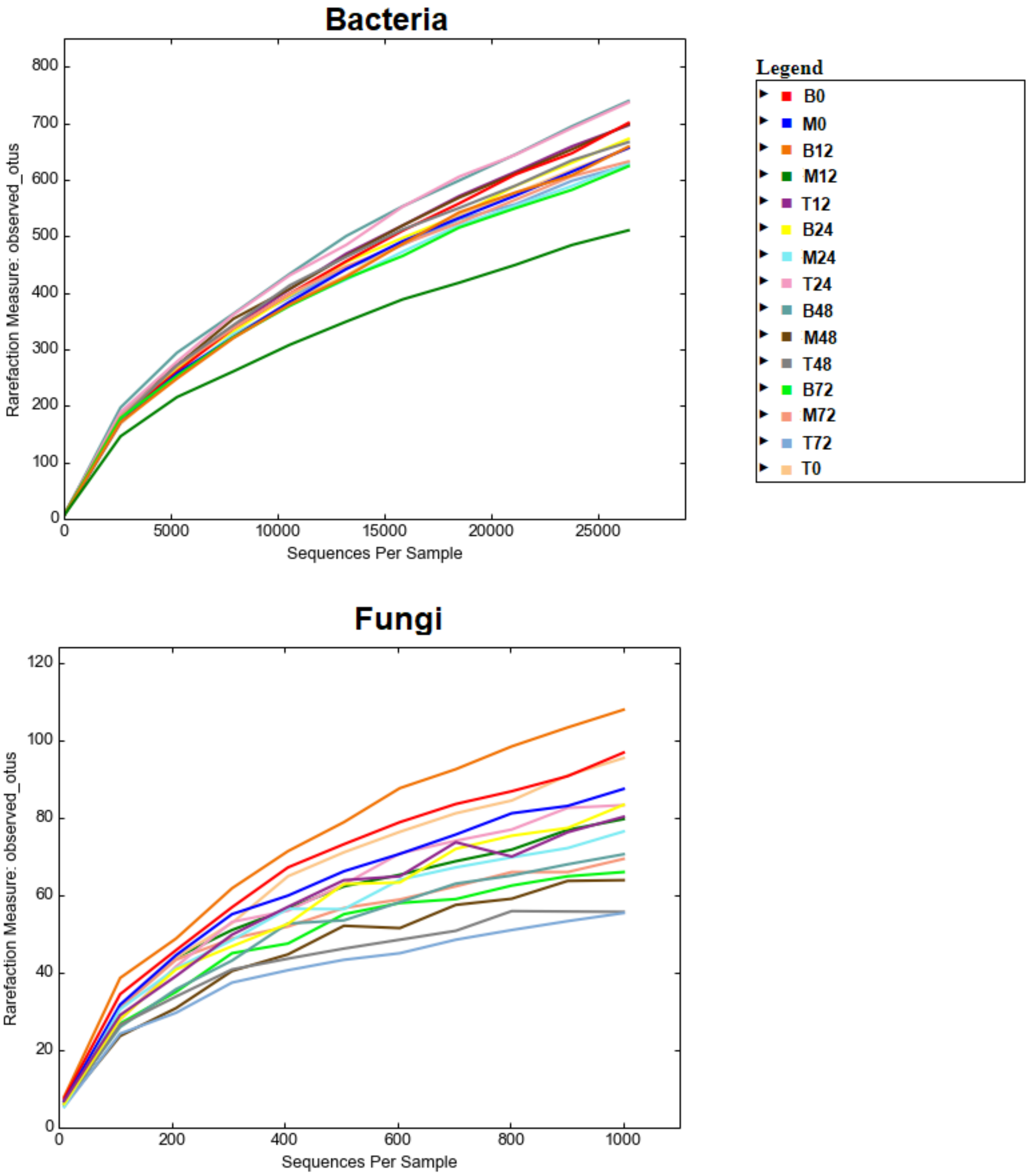

Figure S1: Alpha rarefaction curves of the OTUs (operational taxonomic units) observed from the fermentation liquid fraction samples. (A) Bacterial analysis, (B) Fungal analysis.

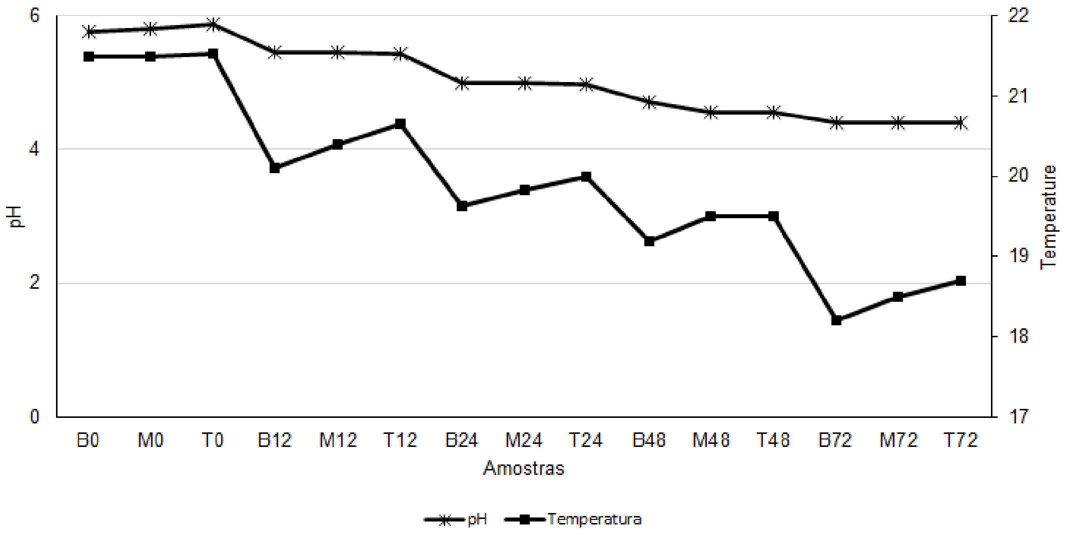

Figure S2: Temperature and pH analysis of the bottom (B), middle (M), and, top (T) layers during spontaneous fermentation. The number after each letter represents the fermentation time (e.g., B0= bottom layer at 0 h).

Table S1: Relative abundance (%) of bacteria and fungi identified during fermentation of Brazilian coffee beans;

Microorganisms	Bacteria														
	**B0**	**M0**	**T0**	**B12**	**M12**	**T12**	**B24**	**M24**	**T24**	**B48**	**M48**	**T48**	**B72**	**M72**	**T72**
Other	0,957625673	0,924161035	0,992397687	1,011105586	1,008736828	1,246301299	0,736855571	0,648057157	0,875672928	0,549213513	0,7913124	0,724453943	0,478236986	0,482854879	0,639705085
Enterobacteriaceae	51,28779941	45,36475481	61,54231347	66,86971656	64,92763772	60,7246237	55,39209415	55,4832541	56,51671634	37,77626601	42,33942251	42,34071069	32,59766698	32,48330756	38,71842134
Erwinia	36,01695948	40,50135736	27,65967133	21,45905851	21,77537682	27,92358163	28,07470897	30,06560251	28,82558166	42,96807593	28,3862278	42,21755352	36,95996381	33,85265382	30,00975821
Enterobacter	2,395282536	2,645410963	1,973414667	1,786010277	1,824062959	2,343046443	1,322758091	1,304082229	1,839144197	1,431937347	1,393214917	2,894193502	1,040488577	1,063789656	1,134121219
Klebsiella	0,00731012	0	0,009104566	0,004143875	0,020008003	0,003216261	0,005117053	0,007967916	0,046209653	0,006637021	0,004209109	0,007244539	0	0,003772304	0,008673967
Providencia	0	0	0	0	0	0	0	0	0,004620965	0	0	0,007244539	0	0	0
Salmonella	0	0	0,002276141	0,002071938	0	0	0	0	0	0	0	0,00362227	0	0,003772304	0
Serratia	0,645727235	1,08011321	0,915008877	0,354301343	0,313458717	0,678631159	0,058846105	0,079679159	0,145560408	0,04977766	0,143109689	0,12315717	0,048469965	0,007544607	0,11926705
Lactobacillaceae	0,173006165	0,213712239	0,252651705	0,120172385	0,123382686	0,06914962	2,5303825	1,508592069	0,732423003	2,404260968	3,830288745	1,467019234	7,286651372	13,4935305	12,30835954
Lactobacillus	0,399619874	0,537168602	0,546273956	0,306646776	0,526877418	0,209056992	3,960598695	3,561658389	1,707446686	8,614853654	11,53295732	4,625638425	12,31137105	6,70715606	6,488127507
Pediococcus	0	0	0	0	0	0,001608131	0,002558526	0	0	0,003318511	0	0	0	0	0
Weissella	0,095031555	0,080864091	0,020485273	0,225841207	0,181739362	0,032162614	0,376103364	0,20450984	0,136318477	0,214043937	0,387237983	0,094179013	0,475005655	0,671470067	0,236365608
Leuconostocaceae	0,494651429	0,508288569	0,186643602	1,085695342	1,205482193	0,34092371	1,565818089	2,71971528	2,395970518	0,721776067	1,633134102	0,583185424	0,594564901	1,433475423	1,097256858
Leuconostoc	3,949901313	2,818691157	0,849000774	3,356539035	5,540549553	2,373600926	2,834847128	1,543119705	2,451422102	2,05913586	4,310127115	1,021480059	2,814489288	3,579916255	1,867071452
Enterococcaceae	0,002436707	0	0	0	0	0	0,017909684	0,005311944	0,004620965	0,001659255	0	0	0	0,003772304	0,002168492
Enterococcus	0,065791077	0,075088084	0,07738881	0,031079065	0,050020008	0,020905699	0,094665473	0,058431383	0,057762066	0,122784894	0,105227713	0,076067664	0,313439106	0,24142744	0,219017673
Lactococcus	0,650600648	0,693120776	0,3072791	2,109232554	1,433906896	1,473047729	2,169630293	1,532495817	2,370555209	1,309152452	3,131576732	1,658999529	1,783694704	2,870723151	3,883768839
Acetobacteraceae	0,384999634	0,635360712	0,901352028	0,070445881	0,156729358	0,358613148	0,061404631	0,084991102	0,140939442	0,53262096	0,416701743	0,326004274	1,689986105	0,720510015	0,379486067
Acetobacter	0,031677185	0,028880032	0,034142122	0	0,005002001	0,020905699	0,005117053	0,007967916	0,004620965	0,004977766	0,042091085	0,021733618	0,029081979	0,022633822	0,006505475
Pseudomonadaceae	0,004873413	0,011552013	0,006828424	0,006215813	0,003334667	0,012865046	0,010234105	0,007967916	0,011552413	0,014933298	0,004209109	0,014489079	0,03231331	0,018861519	0,008673967

Pseudomonas	0,155949219	0,462080518	0,375563345	0,159539201	0,10670935	0,246043998	0,117692209	0,148734429	0,145560408	0,232295746	0,218873643	0,144890789	0,252043817	0,505488702	0,338284723
Chryseobacterium	0,404493287	0,629584705	1,101652479	0,033151003	0,10170735	0,2042326	0,028143789	0,018591804	0,080866893	0,038162873	0,109436821	0,159379867	0,109865253	0,109396809	0,097582132
Aurantimonadaceae	0,024367065	0,005776006	0,011380707	0,024863252	0,003334667	0,025730091	0,012792631	0,002655972	0,016173379	0,011614787	0,008418217	0,014489079	0,009693993	0,015089215	0,052043804
Comamonadaceae	0,004873413	0,057760065	0,066008103	0,010359688	0,015006002	0,038595137	0,007675579	0,007967916	0,018483861	0,011614787	0,021045543	0,021733618	0,006462662	0,022633822	0,073728722
Oxalobacteraceae	0,021930359	0,017328019	0,059179679	0,022791314	0,021675337	0,041811398	0,010234105	0,015935832	0,025415309	0,013274043	0,016836434	0,00362227	0,029081979	0,06035686	0,030358885
Sphingomonadaceae	0,060917664	0,167504188	0,068284245	0,002071938	0,01834067	0,072365882	0,02046821	0,010623888	0,016173379	0,008296277	0,037881977	0,025355888	0,019387986	0,015089215	0,04770682
Acinetobacter	0,036550598	0,127072142	0,036418264	0,047654567	0,040016006	0,059500836	0,023026737	0,039839579	0,018483861	0,011614787	0,021045543	0,083312203	0,016156655	0,026406126	0,021684918
Agrobacterium	0,026803772	0,011552013	0,043246688	0,008287751	0,006669334	0,017689438	0,002558526	0,007967916	0,006931448	0,008296277	0,02946376	0,014489079	0,006462662	0,007544607	0,019516426
Bacteroides	0,065791077	0,063536071	0,081941093	0,020719377	0,01834067	0,028946353	0,010234105	0,037183607	0,10397172	0,031525851	0,037881977	0,036222697	0,016156655	0,045267645	0,117098558
[Barnesiellaceae]	0,00731012	0	0	0	0	0	0	0	0	0	0	0	0	0	0
0319-6A21	0	0	0	0	0	0,001608131	0,002558526	0	0	0	0	0	0	0	0
Aeromonadaceae	0,009746826	0	0	0	0	0	0	0	0	0	0	0	0	0	0
Alcaligenaceae	0,009746826	0,005776006	0	0	0,003334667	0,009648784	0,005117053	0,002655972	0,002310483	0,003318511	0	0	0,006462662	0,003772304	0
Alicyclobacillaceae	0	0	0	0,002071938	0	0	0	0,002655972	0	0	0	0	0	0	0
Bacillaceae	0	0	0	0	0	0	0	0	0,004620965	0	0	0	0	0	0
Bacteriovoracaceae	0	0	0,002276141	0	0	0,001608131	0	0,01327986	0	0	0	0	0	0,007544607	0,006505475
Beijerinckiaceae	0,004873413	0	0	0	0	0	0	0	0	0	0	0	0	0	0
Bifidobacteriaceae	0	0	0	0	0	0	0	0	0	0	0	0	0,006462662	0	0
Bradyrhizobiaceae	0	0	0,002276141	0	0	0	0	0	0	0	0	0	0,006462662	0	0,002168492
Brucellaceae	0	0	0	0	0,001667334	0	0	0	0	0	0	0,007244539	0	0	0
Caulobacteraceae	0,019493652	0,011552013	0	0,004143875	0,005002001	0,009648784	0	0,002655972	0,002310483	0,001659255	0,008418217	0,018111349	0,029081979	0	0,013010951
Cellulomonadaceae	0	0	0	0	0	0,001608131	0,002558526	0	0	0	0	0,00362227	0	0,003772304	0
Chitinophagaceae	0,014620239	0,034656039	0,047798971	0,006215813	0,013338669	0,006432523	0,012792631	0,002655972	0,002310483	0	0	0	0,003231331	0,011316911	0

Christensenellaceae	0,002436707	0	0	0,002071938	0	0	0	0,002655972	0	0	0	0	0	0	0,002168492
Clostridiaceae	0,021930359	0,023104026	0,004552283	0,004143875	0,005002001	0	0,010234105	0,007967916	0,004620965	0,003318511	0,004209109	0	0	0,011316911	0,002168492
Coxiellaceae	0	0	0	0	0	0	0	0	0,004620965	0	0	0	0	0	0
Cytophagaceae	0	0	0	0	0	0,003216261	0	0	0	0	0,004209109	0	0	0	0
D2	0	0	0	0,004143875	0	0	0	0	0	0	0	0	0	0	0
Dermabacteraceae	0	0	0	0	0	0	0	0	0	0,003318511	0	0	0	0,026406126	0
Ellin6075	0,017056946	0	0,013656849	0,002071938	0,006669334	0,009648784	0,017909684	0,010623888	0,016173379	0,003318511	0,008418217	0,007244539	0	0,003772304	0,032527377
Erysipelotrichaceae	0	0	0,002276141	0	0	0	0	0,002655972	0	0	0	0	0	0	0
Erythrobacteraceae	0,002436707	0,005776006	0,002276141	0	0,001667334	0,004824392	0	0	0,006931448	0,001659255	0	0	0	0	0,006505475
FFCH4570	0	0	0	0	0	0	0	0	0	0	0,008418217	0	0	0	0
Flavobacteriaceae	0	0	0,018209132	0	0	0,003216261	0	0	0	0	0	0	0	0	0
Frankiaceae	0	0	0	0	0,001667334	0	0	0	0	0	0	0	0	0	0,002168492
Gaiellaceae	0	0	0	0	0	0	0	0,002655972	0	0	0	0	0,003231331	0,007544607	0
Gemellaceae	0	0	0	0	0	0	0	0	0	0,004977766	0	0	0	0	0
Gemmataceae	0	0	0	0	0	0	0,005117053	0	0	0	0	0	0	0	0
Geodermatophilaceae	0	0	0	0	0	0,004824392	0	0	0,002310483	0	0	0	0	0	0
Helicobacteraceae	0	0	0	0	0	0	0,002558526	0	0,002310483	0,003318511	0	0	0	0	0
Hyphomonadaceae	0	0	0	0	0	0,001608131	0	0	0	0	0,004209109	0	0	0	0
Isosphaeraceae	0,004873413	0	0,006828424	0	0	0,003216261	0	0	0	0	0	0,00362227	0	0	0
Koribacteraceae	0	0	0	0	0,001667334	0,001608131	0,002558526	0	0	0	0	0	0	0	0,004336984
Lachnospiraceae	0,14132898	0,023104026	0,029589839	0,014503564	0,008336668	0,012865046	0,005117053	0,007967916	0,032346757	0,013274043	0,021045543	0	0,012925324	0,026406126	0,054212295
mb2424	0	0	0	0	0,001667334	0	0,007675579	0	0	0	0,004209109	0	0	0	0
Methylobacteriaceae	0,00731012	0,011552013	0,01593299	0,002071938	0,001667334	0,064325228	0	0,005311944	0,013862896	0,001659255	0,046300194	0,00362227	0,006462662	0	0,043369836

Methylocystaceae	0	0	0	0	0	0,030554483	0,002558526	0	0,002310483	0	0	0	0	0	0,015179443
Methylophilaceae	0	0	0	0	0,001667334	0	0	0	0,006931448	0	0	0	0	0	0
Microbacteriaceae	0,002436707	0	0,002276141	0,004143875	0,001667334	0,001608131	0,002558526	0,002655972	0	0,001659255	0,004209109	0,00362227	0,003231331	0	0,019516426
Micrococcaceae	0	0	0	0	0	0,003216261	0	0,002655972	0	0	0,004209109	0	0	0	0
Micromonosporaceae	0	0	0	0	0	0,006432523	0	0	0,002310483	0	0,004209109	0	0	0	0
Moraxellaceae	0,002436707	0,005776006	0	0	0	0,001608131	0	0	0	0	0	0	0	0	0
Nocardioidaceae	0	0	0,002276141	0,002071938	0	0,011256915	0,002558526	0	0,002310483	0,001659255	0	0,00362227	0,006462662	0,015089215	0,04770682
Paenibacillaceae	0	0	0,006828424	0	0	0,006432523	0,002558526	0	0,002310483	0	0	0	0,009693993	0,041495341	0,006505475
Phyllobacteriaceae	0	0,011552013	0	0	0	0,001608131	0	0	0	0	0	0	0	0	0
Pirellulaceae	0	0	0,020485273	0	0	0	0	0	0	0	0	0	0	0	0
Piscirickettsiaceae	0	0	0	0	0,001667334	0	0,007675579	0	0	0	0,004209109	0	0	0	0
Porphyromonadaceae	0,017056946	0,005776006	0,018209132	0,014503564	0,005002001	0,016081307	0	0,061087355	0,013862896	0,001659255	0,008418217	0,010866809	0,012925324	0,033950734	0,034695869
Procabacteriaceae	0	0	0	0	0	0	0	0	0	0	0	0	0,012925324	0	0
PRR-10	0	0	0	0	0	0,003216261	0,005117053	0	0	0	0	0	0	0	0
Rhizobiaceae	0,002436707	0	0	0	0	0	0	0	0	0,001659255	0	0	0,003231331	0	0,002168492
Rhodobacteraceae	0,002436707	0	0,004552283	0	0	0	0	0	0	0	0	0	0	0	0
Rhodocyclaceae	0	0	0,002276141	0,002071938	0	0	0,005117053	0,002655972	0	0	0,004209109	0	0	0,011316911	0
Rhodospirillaceae	0	0	0	0	0	0,001608131	0,007675579	0,002655972	0	0	0,008418217	0	0	0	0,002168492
Ruminococcaceae	0,026803772	0,005776006	0,009104566	0	0	0	0	0	0,009241931	0	0	0,007244539	0	0	0
S24-7	0,053607544	0,011552013	0,011380707	0	0	0	0	0,002655972	0,004620965	0	0	0	0	0	0,021684918
Sinobacteraceae	0	0	0,004552283	0	0	0,008040654	0	0	0,002310483	0	0	0	0	0,007544607	0
Solibacteraceae	0	0	0,002276141	0,002071938	0	0	0	0	0	0	0	0	0	0	0
Sphingobacteriaceae	0,00731012	0,011552013	0,047798971	0,018647439	0,003334667	0,014473176	0,005117053	0,007967916	0,006931448	0,013274043	0,016836434	0,014489079	0,003231331	0	0,067223246

Syntrophobacteraceae	0	0,005776006	0	0	0	0	0,002558526	0	0	0	0,004209109	0	0	0,003772304	0
Veillonellaceae	0,012183533	0,005776006	0,009104566	0,014503564	0,010004002	0,009648784	0,005117053	0	0,011552413	0,008296277	0,025254651	0,00362227	0,016156655	0	0,002168492
Xanthomonadaceae	0,00731012	0	0	0,002071938	0,001667334	0,017689438	0,002558526	0,007967916	0,002310483	0,011614787	0,012627326	0,00362227	0,071089282	0,026406126	0
[Prevotella]	0	0	0	0	0	0	0	0	0	0,003318511	0	0	0	0	0
[Ruminococcus]	0,002436707	0	0	0	0	0	0	0	0	0	0	0	0	0	0,002168492
A17	0	0	0	0	0	0	0	0,002655972	0,006931448	0	0	0	0	0	0
Achromobacter	0	0	0	0	0	0,006432523	0	0,002655972	0	0,003318511	0	0	0	0	0
Acidaminobacter	0,004873413	0	0	0,002071938	0	0	0	0	0,002310483	0	0	0	0	0	0
Acidovorax	0,002436707	0	0,006828424	0	0	0,003216261	0	0	0,006931448	0	0,016836434	0,00362227	0	0	0
Actinomyces	0	0	0	0	0	0	0	0	0	0,003318511	0	0	0	0	0
Actinomycetospora	0,00731012	0,005776006	0,013656849	0,006215813	0	0,003216261	0	0,002655972	0	0	0,008418217	0	0,003231331	0	0
Aerococcus	0	0	0	0	0	0	0	0	0	0,001659255	0	0	0,003231331	0,007544607	0,006505475
Aeromicrobium	0	0	0	0	0	0	0,007675579	0	0	0	0	0	0	0,007544607	0
Aggregatibacter	0	0	0	0	0	0	0	0	0	0,003318511	0	0	0	0	0
Akkermansia	0,004873413	0	0	0	0	0	0	0	0	0	0	0	0	0	0
Allochromatium	0	0	0	0	0	0,001608131	0	0	0	0	0	0	0	0	0,004336984
Aminobacter	0	0,011552013	0,002276141	0	0	0,001608131	0	0	0	0	0	0	0	0	0
Ammoniphilus	0	0	0	0	0	0,001608131	0	0	0	0	0	0	0	0	0,002168492
Amycolatopsis	0	0	0	0	0	0	0	0	0	0	0,008418217	0	0	0	0
Anaerofilum	0	0	0,009104566	0	0	0	0	0	0	0	0	0	0	0	0
Anaerolinea	0	0	0	0	0	0,001608131	0	0	0	0	0,004209109	0	0	0	0
Anaeromusa	0	0	0	0	0	0,003216261	0	0,002655972	0	0,003318511	0	0	0	0	0
Anoxybacillus	0	0	0	0	0	0	0	0	0,004620965	0	0	0	0	0	0

Aquicella	0	0	0,006828424	0	0	0	0	0	0	0	0	0	0	0	0
Arcobacter	0,00731012	0,028880032	0,027313698	0	0,010004002	0,020905699	0,012792631	0,007967916	0,013862896	0,048118404	0,004209109	0,018111349	0,012925324	0,056584556	0
Arsenicicoccus	0	0	0	0	0	0,006432523	0	0	0	0	0	0	0	0	0
Arthrobacter	0	0	0	0	0	0,003216261	0	0	0	0,001659255	0	0,00362227	0	0	0
Aurantimonas	0,00731012	0	0,009104566	0	0	0,008040654	0	0	0	0	0	0,00362227	0	0,026406126	0,032527377
Azospira	0,002436707	0	0	0	0,001667334	0	0	0	0	0,001659255	0	0	0	0	0
Bacillus	0	0	0	0	0,003334667	0	0	0,002655972	0,030036275	0,028207341	0,004209109	0	0,009693993	0	0
Bdellovibrio	0,004873413	0	0,002276141	0	0,001667334	0,009648784	0	0	0	0	0,004209109	0,007244539	0	0	0
Bifidobacterium	0,009746826	0,005776006	0	0	0	0	0	0	0,002310483	0	0	0	0	0	0
Blastomonas	0	0	0,002276141	0	0	0,001608131	0,005117053	0	0	0	0	0	0	0	0,006505475
Blvii28	0	0	0	0	0	0	0,005117053	0	0	0	0	0	0	0,018861519	0
Brachybacterium	0	0	0,006828424	0	0	0	0	0	0,002310483	0,004977766	0,004209109	0,00362227	0,003231331	0,026406126	0,021684918
Brevibacterium	0	0	0	0	0	0	0	0	0	0	0	0	0,006462662	0	0
Brevundimonas	0,029240479	0,011552013	0,002276141	0	0	0,001608131	0	0	0	0	0	0	0	0	0
Burkholderia	0,014620239	0,017328019	0,004552283	0,002071938	0,001667334	0,008040654	0,002558526	0	0,004620965	0,003318511	0,025254651	0,010866809	0	0,052812252	0,026021902
Candidatus Koribacter	0	0	0,004552283	0	0	0,001608131	0	0	0	0	0	0	0	0	0,008673967
Candidatus Portiera	0,002436707	0,005776006	0,011380707	0	0,003334667	0,001608131	0,002558526	0	0	0,009955532	0	0,00362227	0,019387986	0,037723037	0,006505475
Candidatus Solibacter	0	0	0	0	0	0	0,002558526	0	0,002310483	0	0	0	0	0	0,002168492
Carnobacterium	0	0	0	0	0	0	0	0	0	0,001659255	0	0,00362227	0	0,052812252	0
Cellvibrio	0	0	0	0	0	0,003216261	0	0	0	0	0	0	0	0	0
Chthoniobacter	0	0	0,004552283	0	0	0	0	0	0	0	0	0	0	0	0
Citrobacter	0,051170837	0,034656039	0,029589839	0,043510691	0,06502601	0,049852052	0,017909684	0,021247776	0,057762066	0,028207341	0,042091085	0,028978158	0,06462662	0,018861519	0,026021902
Cloacibacterium	0,004873413	0	0,018209132	0,012431626	0,006669334	0,001608131	0,005117053	0,055775411	0,060072549	0	0,008418217	0,010866809	0	0	0

Clostridium	0,121835327	0,716224802	0,072836528	0,05387038	0,025010004	0,020905699	0,023026737	0,01327986	0,050830619	0,016592553	0,008418217	0,014489079	0,012925324	0,022633822	0,075897213
Collimonas	0	0	0	0	0	0	0,002558526	0	0	0,003318511	0	0	0,003231331	0	0
Collinsella	0,009746826	0	0	0	0	0	0,002558526	0	0,002310483	0	0	0	0	0	0
Comamonas	0,112088501	0,103968116	0,034142122	0,018647439	0,025010004	0,043419529	0,033260842	0,069055271	0,023104827	0,036503617	0,021045543	0,224580722	0,061395289	0,022633822	0,039032853
Coprococcus	0,031677185	0,017328019	0,011380707	0	0,005002001	0,003216261	0,010234105	0,005311944	0,013862896	0,006637021	0,016836434	0,014489079	0,03231331	0,022633822	0,010842459
Curtobacterium	0,012183533	0	0,011380707	0	0,003334667	0,008040654	0	0	0	0,008296277	0,012627326	0	0,016156655	0,003772304	0,032527377
Cytophaga	0	0	0	0,008287751	0	0	0	0	0	0	0	0	0	0	0
DA101	0	0	0	0	0	0	0,005117053	0	0	0	0	0	0	0	0
Deinococcus	0	0	0,006828424	0	0	0	0	0	0	0	0	0	0	0	0
Delftia	0,009746826	0,017328019	0,013656849	0,03522294	0,005002001	0,004824392	0	0,005311944	0	0,006637021	0	0,010866809	0,003231331	0,018861519	0,054212295
Desulfovibrio	0	0	0	0	0,001667334	0	0	0,002655972	0,004620965	0,001659255	0	0	0	0	0
Devosia	0	0	0,004552283	0	0,001667334	0,003216261	0	0	0	0	0	0,00362227	0	0,003772304	0
Dorea	0	0	0	0	0,011671335	0	0	0	0	0	0	0	0	0	0
Dyadobacter	0,00731012	0	0	0	0	0	0	0	0	0	0	0,00362227	0	0	0
Dysgonomonas	0,041424011	0,051984058	0,100150225	0,002071938	0,011671335	0,04502766	0,002558526	0,015935832	0,023104827	0,031525851	0,067345736	0,115912631	0,025850648	0,026406126	0,004336984
Ellin506	0	0	0	0	0	0,004824392	0	0	0	0	0	0	0	0	0
Enhydrobacter	0,029240479	0,075088084	0,050075113	0,070445881	0,02334267	0,083622797	0,010234105	0,002655972	0,023104827	0,014933298	0,046300194	0,025355888	0	0,003772304	0,026021902
Epulopiscium	0	0	0	0	0	0,003216261	0	0	0	0	0	0	0	0	0
Erysipelothrix	0,004873413	0,017328019	0,006828424	0	0	0,003216261	0,002558526	0,002655972	0	0	0,008418217	0,00362227	0,025850648	0	0,028190394
Exiguobacterium	0	0	0	0	0	0	0	0	0	0,014933298	0	0	0,006462662	0,045267645	0,008673967
Faecalibacterium	0,041424011	0,005776006	0	0	0	0	0	0	0,004620965	0	0	0	0	0	0,002168492
Fimbriimonas	0	0,005776006	0,002276141	0	0	0	0,005117053	0	0,002310483	0	0	0	0	0,007544607	0,019516426
Flavisolibacter	0	0	0	0	0	0,003216261	0	0	0	0	0	0	0	0	0

Flavobacterium	0,004873413	0,057760065	0,01593299	0,002071938	0,005002001	0,004824392	0,007675579	0,018591804	0,009241931	0,004977766	0	0,025355888	0	0,011316911	0
Fluviicola	0	0	0,004552283	0,008287751	0	0,001608131	0,015351158	0	0	0,001659255	0	0,010866809	0	0,011316911	0,058549279
Friedmanniella	0	0	0	0	0	0,006432523	0	0	0	0	0	0	0	0	0
Fusibacter	0,00731012	0	0	0	0	0	0	0,002655972	0,002310483	0	0	0	0	0	0
Gallionella	0	0	0	0	0	0	0	0	0	0	0	0	0	0	0,017347935
Gemmata	0	0	0	0	0	0,003216261	0	0,002655972	0	0	0	0	0,003231331	0	0
Geobacter	0	0	0	0	0,003334667	0	0	0	0	0	0	0	0	0	0
Geodermatophilus	0	0	0,002276141	0	0	0,001608131	0	0	0	0	0	0	0	0	0
Giesbergeria	0,002436707	0,011552013	0,006828424	0,008287751	0	0	0,002558526	0,002655972	0	0	0	0	0	0,011316911	0
Granulicatella	0	0	0	0	0	0	0	0	0	0,008296277	0	0	0	0	0
Haemophilus	0	0	0	0	0	0	0	0	0	0,003318511	0	0	0	0	0
Hymenobacter	0,009746826	0,011552013	0,059179679	0,004143875	0,008336668	0,014473176	0,005117053	0,010623888	0,048520136	0,031525851	0,02946376	0,050711776	0,03231331	0,03017843	0,164805378
Janibacter	0	0	0	0	0	0	0	0	0	0,001659255	0,004209109	0,00362227	0,019387986	0,007544607	0
Janthinobacterium	0,00731012	0	0,013656849	0,002071938	0	0,004824392	0,002558526	0,002655972	0	0,001659255	0	0,00362227	0,003231331	0,011316911	0
K82	0	0	0	0	0	0	0	0	0,004620965	0	0	0	0	0	0,002168492
Kaistobacter	0,00731012	0,017328019	0,038694405	0	0,005002001	0,033770745	0,023026737	0,005311944	0,006931448	0,008296277	0	0,010866809	0,003231331	0,018861519	0,006505475
KD1-23	0	0	0,004552283	0	0	0	0	0,002655972	0	0	0	0	0	0	0
Kineococcus	0	0	0	0	0,003334667	0	0	0	0	0	0	0	0	0	0
Kocuria	0	0	0	0	0,001667334	0	0	0	0,020794344	0	0	0	0	0	0
Lachnospira	0,00731012	0	0	0	0	0	0	0	0	0	0	0	0	0	0
Lampropedia	0	0	0,009104566	0	0	0	0	0	0	0	0	0	0	0	0
Legionella	0	0	0	0	0	0,003216261	0	0	0	0	0	0	0	0	0
Listeria	0	0	0	0	0	0	0	0	0	0	0	0	0	0,011316911	0

Luteibacter	0	0,005776006	0	0	0	0,011256915	0	0,002655972	0,009241931	0,001659255	0,012627326	0,00362227	0	0	0
Luteimonas	0	0,005776006	0,002276141	0	0	0	0	0	0	0,001659255	0	0,007244539	0	0	0
Lysobacter	0	0,005776006	0	0	0	0,006432523	0,012792631	0	0,002310483	0	0	0	0	0	0
Magnetospirillum	0	0	0	0	0	0	0,005117053	0	0,002310483	0,013274043	0,004209109	0	0,003231331	0	0
Megasphaera	0	0	0,002276141	0	0,003334667	0	0	0	0	0	0	0	0	0,003772304	0,002168492
Methylibium	0	0	0,004552283	0	0	0,001608131	0	0	0	0	0	0	0	0	0
Methylobacterium	0,073101196	0,121296136	0,152501479	0,076661694	0,026677338	0,192975685	0,030702315	0,031871663	0,067003997	0,044799894	0,109436821	0,057956315	0,061395289	0,067901467	0,201669739
Microbacterium	0,004873413	0	0	0	0	0,003216261	0	0	0	0,001659255	0,004209109	0	0	0	0
Mitsuaria	0	0	0	0	0	0,004824392	0	0	0	0	0	0	0	0	0
Mycobacterium	0	0	0	0	0	0,006432523	0	0	0	0	0,004209109	0	0	0,007544607	0
Neisseria	0	0	0	0	0	0	0	0	0	0,003318511	0	0	0	0	0
Nitrospira	0	0	0	0	0	0,004824392	0,002558526	0	0	0	0,004209109	0	0	0	0
Nocardioides	0	0	0	0	0	0	0	0	0	0	0	0	0	0,011316911	0
Novispirillum	0,002436707	0	0	0	0,001667334	0	0	0	0	0	0	0	0,003231331	0	0
Novosphingobium	0,00731012	0,017328019	0,013656849	0	0,010004002	0,019297569	0,002558526	0,005311944	0,002310483	0,008296277	0,025254651	0	0,003231331	0	0,013010951
Ochrobactrum	0,012183533	0,034656039	0,006828424	0	0,001667334	0,012865046	0	0,002655972	0	0	0,025254651	0,018111349	0,019387986	0,033950734	0,019516426
Opitutus	0	0	0	0	0	0,001608131	0,002558526	0	0	0	0	0	0	0	0
Oscillospira	0,004873413	0	0	0,002071938	0	0	0	0	0,004620965	0	0	0	0,003231331	0	0,002168492
Paenibacillus	0,00731012	0	0,01593299	0	0,001667334	0	0,002558526	0,002655972	0	0	0	0	0	0,018861519	0,002168492
Paludibacter	0	0,011552013	0	0,006215813	0,001667334	0	0,005117053	0,007967916	0,006931448	0,003318511	0	0,010866809	0	0	0
Pantoea	0,021930359	0,034656039	0,025037556	0,022791314	0,021675337	0,024121961	0,030702315	0,021247776	0,048520136	0,033185107	0,02946376	0,014489079	0,029081979	0,052812252	0,017347935
Paracoccus	0,00731012	0	0	0	0,001667334	0	0	0,002655972	0,006931448	0	0	0	0,006462662	0	0,013010951
Pedobacter	0,00731012	0,017328019	0,031865981	0,002071938	0	0,004824392	0,002558526	0,002655972	0,006931448	0,003318511	0,012627326	0,054334046	0,016156655	0,045267645	0,02385341

Perlucidibaca	0	0	0	0	0	0,036987006	0	0	0	0	0,025254651	0	0	0	0
Pigmentiphaga	0	0	0	0,002071938	0	0,001608131	0	0	0	0	0	0	0	0	0
Planctomyces	0	0	0	0	0	0	0,005117053	0	0	0	0	0	0	0	0
Plesiocystis	0	0	0	0	0	0	0,002558526	0	0	0	0	0	0	0,003772304	0
Prauseria	0	0	0	0	0,001667334	0	0	0	0	0	0	0	0	0	0,002168492
Prevotella	0,124272034	0,306128343	0,289069969	0,15125145	0,056689342	0,109352888	0,030702315	0,021247776	0,067003997	0,09955532	0,046300194	0,032600427	0,180954535	0,207476706	0,140951968
Propionibacterium	0	0	0	0	0	0	0	0	0,002310483	0,001659255	0	0	0	0	0
Proteus	0	0	0	0	0	0,003216261	0,002558526	0	0	0,001659255	0	0,00362227	0,003231331	0	0,002168492
Pseudoclavibacter	0	0	0,002276141	0	0	0	0	0	0	0	0	0	0	0	0,002168492
Pseudonocardia	0	0	0	0	0	0	0	0,002655972	0	0	0,008418217	0,010866809	0	0	0
Rhodobacter	0	0	0,006828424	0	0	0	0	0	0	0	0	0	0	0	0
Rhodococcus	0	0	0	0	0	0,006432523	0	0	0	0	0	0	0	0	0
Ruminococcus	0,034113892	0	0	0	0,001667334	0	0	0	0,013862896	0	0	0	0	0	0,002168492
Sedimentibacter	0	0	0	0	0	0	0	0,005311944	0	0	0	0	0	0	0
Segetibacter	0,009746826	0	0	0,010359688	0	0	0	0	0	0	0	0	0	0	0
Selenomonas	0	0	0	0	0	0	0	0	0	0,003318511	0	0	0	0	0
Simplicispira	0,002436707	0	0,013656849	0	0	0,001608131	0	0	0,002310483	0,003318511	0	0	0	0	0
SMB53	0,004873413	0	0	0	0	0	0	0	0	0	0	0	0	0	0
Sphingobacterium	0,146202393	0,09819211	0,109254791	0,037294878	0,005002001	0,02251383	0,017909684	0,268253167	0,016173379	0,014933298	0,058927519	0,217336183	0,045238634	0,064129164	0,028190394
Sphingobium	0,012183533	0	0,009104566	0	0,008336668	0,009648784	0,002558526	0	0	0,019911064	0	0	0,006462662	0,026406126	0,028190394
Sphingomonas	0,06335437	0,103968116	0,157053762	0,122244323	0,02334267	0,119001672	0,017909684	0,02655972	0,067003997	0,044799894	0,037881977	0,119534901	0,077551944	0,101852201	0,286240919
Spirosoma	0	0,011552013	0,070560386	0	0,001667334	0,001608131	0	0	0,013862896	0,003318511	0,021045543	0,00362227	0,006462662	0,007544607	0,032527377
Sporolactobacillus	0,009746826	0,132848149	0,011380707	0,012431626	0,006669334	0,054676444	0,012792631	0,002655972	0,009241931	0,013274043	0,016836434	0	0	0,003772304	0,002168492

Sporotomaculum	0	0	0	0,004143875	0	0	0	0	0	0	0	0	0	0	0
Staphylococcus	0	0	0	0	0	0	0	0	0	0	0	0	0	0,007544607	0
Stenotrophomonas	0,00731012	0,005776006	0,004552283	0,010359688	0,001667334	0,024121961	0	0	0,009241931	0,02488883	0,008418217	0,018111349	0,006462662	0,015089215	0,086739673
Steroidobacter	0,002436707	0	0	0	0	0,003216261	0,002558526	0	0	0	0	0	0	0	0
Streptococcus	0,004873413	0,005776006	0	0	0	0	0	0	0	0,044799894	0	0	0	0	0
Sulfurospirillum	0	0	0,006828424	0	0	0	0	0,010623888	0,013862896	0,006637021	0,004209109	0,00362227	0,006462662	0,011316911	0,036864361
Sutterella	0,014620239	0	0	0	0	0	0	0,002655972	0	0	0	0	0	0	0
Swaminathania	0	0	0,002276141	0	0	0,001608131	0	0	0	0	0	0	0	0	0
Symbiobacterium	0	0	0	0,008287751	0	0	0	0,002655972	0	0	0	0	0	0	0
Thermomonas	0	0	0	0	0	0	0	0	0	0	0,008418217	0	0	0	0
Thiobacillus	0	0	0	0	0	0,003216261	0,002558526	0	0	0	0,008418217	0	0	0,003772304	0
Trabulsiella	0,019493652	0	0,020485273	0,002071938	0,011671335	0,017689438	0,010234105	0,005311944	0,011552413	0,001659255	0,008418217	0,00362227	0,009693993	0	0,010842459
Trichococcus	0	0	0	0	0	0	0	0	0	0,003318511	0	0	0	0	0
Turicibacter	0,004873413	0	0	0	0	0	0	0	0	0	0	0	0	0	0
vadinHB04	0	0	0	0	0	0,004824392	0	0	0	0	0,008418217	0	0	0	0
Variovorax	0	0	0,004552283	0	0	0,003216261	0	0	0	0	0	0	0	0	0
Veillonella	0,002436707	0	0	0	0	0	0	0	0	0,013274043	0	0	0	0	0
Wautersiella	0	0,005776006	0	0,018647439	0,071695345	0	0	0,042495551	0,32115709	0,004977766	0,004209109	0	0	0	0,019516426
Xenorhabdus	0,002436707	0,005776006	0	0,002071938	0	0	0,002558526	0	0	0	0	0	0	0	0
Zoogloea	0	0	0	0	0,001667334	0	0	0	0	0	0	0	0,006462662	0	0,015179443

**Microorganisms**	**Fungi**													
	**B0**	**M0**	**T0**	**B12**	**M12**	**T12**	**B24**	**M24**	**T24**	**B48**	**M48**	**T48**	**B72**	**M72**	**T72**
Nectriaceae	31,79723502	31,35634419	37,21428571	24,73246136	34,89604566	25,43815088	51,78303866	49,57898958	53,28366987	26,61115134	34,61683654	50,84637496	46,67303407	42,11145997	46,68794892

Mycosphaerellaceae	2,534562212	2,430724356	1,142857143	1,902497027	1,182225846	1,066802134	0,689241834	1,623897354	1,50950794	0,615496017	0,20385051	0,862344299	0,270614454	2,276295133	2,075019952
Trichosporonaceae	1,900921659	1,263976665	1,5	1,783590963	2,609050143	0,96520193	0,71920887	0,741780273	1,274259949	0,579290369	0,792751982	0,606834877	0,668576886	4,081632653	1,356743815
Tremellales	0,806451613	3,354399611	2,642857143	2,615933413	1,182225846	1,371602743	1,798022176	1,002405774	0,960595962	1,013758146	1,834654587	1,149792399	0,620821394	1,628728414	0,03990423
Alternaria	5,011520737	5,930967428	4,285714286	2,615933413	2,935181411	2,489204978	1,947857357	3,047313553	2,117231915	0,687907314	1,67610419	4,982433727	0,891435848	4,238618524	3,471667997
Aureobasidium	0,460829493	0,923675255	0,571428571	1,426872771	0,224215247	0,292100584	0,569373689	0,300721732	0,470495981	0,108616944	0,279350698	0,127754711	0,397962432	0,412087912	0,359138069
Candida	0,921658986	0,777831794	0,428571429	1,664684899	4,443538524	0,571501143	0,149835181	1,864474739	0,352871986	1,593048516	1,381653454	0,606834877	3,311047437	2,06043956	0,399042298
Chaetomella	1,900921659	0,097228974	0	0	0,020383204	0	0	0	0	0,072411296	0,007550019	0	0,015918497	0	0
Cladosporium	7,02764977	8,26446281	8,5	9,750297265	4,891969018	4,152908306	4,465088403	3,869286287	4,332483827	2,353367125	5,790864477	2,906419674	3,215536453	3,198587127	4,269752594
Colletotrichum	2,880184332	5,05590666	6,357142857	5,469678954	5,707297187	2,311404623	1,618219958	3,287890938	2,195647912	2,136133237	1,27595319	2,746726286	1,671442216	3,375196232	7,82122905
Didymella	9,735023041	6,174039864	9,357142857	3,567181926	6,15572768	3,314706629	4,075516931	3,408179631	5,077435797	33,20057929	0,989052473	3,481315874	2,148997135	3,296703297	4,588986433
Fusarium	3,571428571	0,972289742	2,571428571	1,070154578	1,161842642	1,371602743	0,389571471	2,205292702	2,195647912	7,784214337	1,019252548	1,117853721	2,849411016	1,962323391	4,509177973
Glomerella	1,728110599	2,139037433	1,928571429	0,951248514	1,691805952	0,93980188	1,798022176	1,06255012	1,195843952	3,548153512	0,792751982	1,181731076	0,986946832	4,297488226	3,431763767
Kazachstania	0,576036866	0,631988333	0,428571429	1,783590963	0,631879331	15,13843028	1,138747378	1,042502005	2,156439914	1,158580739	10,19252548	1,022037688	2,180834129	0,686813187	0,478850758
Kodamaea	0,17281106	0	0,928571429	0	2,833265389	0,787401575	0,599340725	0,501202887	0,019603999	0,18102824	0	0,031938678	0	0	0,997605746
Penicillium	0,633640553	1,409820126	0,428571429	0,237812128	1,121076233	0,139700279	0,179802218	0,641539695	0,294059988	0,687907314	0,015100038	0,095816033	0	0,35321821	0,159616919
Pestalotiopsis	0,230414747	0,194457948	0,214285714	0,118906064	0,67264574	0,228600457	0,389571471	0,521251002	0,274455989	0,615496017	0	0,542957522	0,206940465	1,766091052	0,678371907
Saccharomyces	0,057603687	0,097228974	0	0,118906064	0,040766408	0,038100076	0,059934073	0,140336808	0,156831994	0,072411296	0,249150623	0,894282977	0,238777459	0,941915228	1,037509976
Strelitziana	0,576036866	0,097228974	0,5	1,070154578	2,833265389	0,431800864	0,119868145	0,781876504	0,294059988	0,253439537	0,347300868	0,031938678	0,334288443	0,824175824	0,03990423
Torulaspora	1,843317972	1,215362178	0,642857143	3,68608799	1,386057888	16,39573279	2,637099191	1,764234162	3,352283866	1,556842867	16,69309173	2,044075375	2,626552053	1,157770801	0,199521149
unidentified	4,320276498	4,958677686	3,214285714	6,896551724	2,772115777	1,765303531	3,775846569	4,711307137	4,959811802	2,353367125	5,269913175	3,321622485	2,722063037	3,335949765	1,835594573
Wickerhamomyces	0,34562212	0,048614487	0,214285714	1,307966706	0	0,457200914	0,059934073	0,080192462	0,725347971	0,144822592	0,27180068	0	0,095510984	0,667189953	0
Other	16,30184332	19,6402528	13	21,52199762	18,263351	16,5481331	17,86035361	16,13873296	10,21368359	11,07892831	12,7595319	17,88565953	24,03693091	14,14835165	11,93136472
Agaricaceae	0	0	0	0	0	0	0	0	0	0	0	0,031938678	0	0	0

Amphisphaeriaceae	0	0	0,071428571	0	0	0	0	0	0	0	0	0	0	0	0
Bionectriaceae	0	0	0	0	0	0	0,059934073	0	0	0	0	0	0	0	0
Botryosphaeriaceae	0	0	0	0,237812128	0	0	0	0	0	0	0	0	0	0	0
Chaetomiaceae	0	0	0	0	0	0	0	0	0	0	0	0,063877355	0	0	0
Diaporthaceae	0	0	0,071428571	0	0	0	0	0	0	0	0	0	0	0	0
Dothioraceae	0	0	0	0	0	0	0	0	0	0	0	0	0,015918497	0	0
Glomerellaceae	0	0	0,071428571	0	0	0	0	0,080192462	0	0	0	0	0	0	0
Herpotrichiellaceae	0	0	0	0	0	0	0	0	0	0	0,007550019	0	0	0	0
Lophiostomataceae	0	0	0	0	0	0,012700025	0	0	0	0	0	0	0	0	0
Pezizomycotina	0	0	0	0	0	0	0,089901109	0	0	0	0	0	0	0,039246468	0
Pleosporales	0	0,097228974	0	0,118906064	0,244598451	0,050800102	0,059934073	0,100240577	0,039207998	0,108616944	0	0	0	0,019623234	0
Saccharomycetaceae	0	0	0	0	0	0,203200406	0	0	0,235247991	0	0,868252171	0,223570744	0,222858962	0	0
Saccharomycetales	0,057603687	0	0	0	0	0	0,179802218	0	0,235247991	0	0,113250283	0	0,015918497	0	0
Sporormiaceae	0	0	0	0	0	0	0	0	0	0	0	0	0	0	0,03990423
Teratosphaeriaceae	0	0	0	0	0	0	0,029967036	0	0,019603999	0	0	0	0	0	0
Trichocomaceae	0,17281106	0	0,071428571	0,118906064	0,020383204	0	0,029967036	0,060144346	0	0,108616944	0	0	0	0,039246468	0
Valsaceae	0	0	0	0	0	0	0,149835181	0	0,235247991	0	0	0,766528266	0,620821394	0,078492936	0,758180367
Acremonium	0	0	0	0	0	0	0,179802218	0	0	0	0	0	0	0	0
Apiotrichum	0,288018433	0,097228974	0	0,356718193	0,061149613	0,063500127	0,029967036	0,040096231	0,117623995	0,036205648	0,052850132	0	0,143266476	0,274725275	0,159616919
Arnium	0	0	0,071428571	0	0	0	0	0	0	0	0	0	0	0	0
Aspergillus	0,115207373	0,194457948	0,214285714	0,475624257	0,285364859	0,076200152	0,539406653	0,020048115	0,411683984	0,072411296	0	0,734589588	0,875517351	0,058869702	0,319233839
Beauveria	0,057603687	0	0	0	0	0	0,029967036	0,100240577	0	0	0	0	0	0,039246468	0
Bipolaris	0,403225806	0,048614487	0	0,237812128	0,14268243	0,127000254	0,119868145	0,120288693	0	0,434467777	0	0	0,493473416	0,470957614	0,11971269

Buckleyzyma	0	0	0	0	0	0,088900178	0	0	0,058811998	0	0	0	0	0	0
Bullera	0	0,145843461	0,071428571	0,118906064	0	0	0	0	0	0	0,354850887	0	0	0	0
Cercospora	0	0	0	0	0	0,012700025	0	0	0	0	0	0	0	0,039246468	0
Chaetomium	0	0	0,142857143	0	0,020383204	0	0	0	0,019603999	0	0	0	0,015918497	0	0
Coprinellus	0	0	0,214285714	0	0	0	0	0	0	0	0	0	0	0	0,07980846
Curvularia	0,115207373	0	0	0	0	0	0	0	0	0	0	0	0	0,019623234	0
Cutaneotrichosporon	0	0	0,785714286	0,951248514	0	0,050800102	0,329637399	0	0,156831994	0,036205648	0,234050585	0	0	0	0,598563448
Diaporthe	0	0	0,071428571	0	0	0,012700025	0	0,300721732	0	0,108616944	0	0	0	0,058869702	0
Dioszegia	0	0	0	0	0	0,355600711	0	0	0	0	0,007550019	0	0	0	0
Diplodia	0	0	0	0	0	0	0	0	0	0	0	0	0	0	0,03990423
Dothiorella	0	0	0	0,356718193	0	0	0	0	0	0	0	0	0	0	0
Endomelanconiopsis	0	0	0	0	0	0,012700025	0	0	0	0	0	0	0	0	0
Exophiala	0	0	0	0	0	0	0	0	0	0	0	0	0	0,196232339	0
Filobasidium	0	0	0	0	0	0	0	0	0,019603999	0	0	0	0	0	0
Geosmithia	0	0	0	0	0	0	0	0	0	0	0	0	0,015918497	0	0
Hannaella	0,576036866	0,437530384	0	0,118906064	0	0,063500127	0	0	0,019603999	0	0,302000755	0	0	0	0
Hanseniaspora	0,17281106	0,194457948	0	0,118906064	0,122299225	0,33020066	0,299670363	0,40096231	0,156831994	0,108616944	0,347300868	0,095816033	0,477554919	0,058869702	0,239425379
Heterogastridium	0	0	0	0	0	0	0	0	0	0	0,052850132	0	0	0	0
Isaria	0	0	0,071428571	0	0	0	0	0	0,117623995	0,036205648	0	0	0	0	0
Issatchenkia	0,8640553	0,048614487	0,857142857	0,237812128	0	0,177800356	0,119868145	0	0	0	0,226500566	0	0,191021968	0	0,159616919
Kluyveromyces	0	0	0	0	0	0	0	0,040096231	0	0	0	0	0	0	0
Kwoniella	0	0,145843461	0	0	0,264981655	0	0,179802218	0	0	0	0,030200076	0,191632066	0	0	0
Meira	0,288018433	0,777831794	0,071428571	0,356718193	0,346514472	0,419100838	0,149835181	0	0,215643991	0,217233888	0,143450359	0,063877355	0,366125438	0,431711146	0,798084597

Meyerozyma	0,17281106	0,194457948	0,357142857	0,951248514	0,101916021	0,381000762	0,059934073	0,100240577	0,235247991	0,217233888	0,27180068	0,127754711	0,095510984	0,706436421	0
Microdochium	0	0	0	0	0	0	0	0	0	0	0,007550019	0	0	0	0
Mortierella	0,230414747	0	0	0,237812128	0,020383204	0	0	0,020048115	0	0	0	0,031938678	0,015918497	0,019623234	0
Mucor	0	0	0	0	0	0,609601219	0	0,120288693	0	0	0,279350698	0	0	0	0
Nigrospora	0	0	0,071428571	0	0,163065634	0	0	0	0	0	0	0	0	0,019623234	0
Ochroconis	0,17281106	0,145843461	0,428571429	0	0,407664085	0,063500127	0,059934073	0,040096231	0,098019996	0	0	0	0,079592486	0	0
Periconia	0	0	0	0	0	0,203200406	0	0	0	0	0	0	0	0	0
Pichia	0	0,145843461	0	0,356718193	0	0	0	0	0,019603999	0	0,211400529	0	0	0	0
Purpureocillium	0	0	0	0	0	0,076200152	0	0	0,019603999	0	0	0	0	0	0
Rhizopus	0	0	0	0	0	0	0	0	0	0	0	0	0,015918497	0	0
Rhodosporidiobolus	0	0	0	0	0	0	0	0	0	0	0	0	0,015918497	0,412087912	0,319233839
Rhodotorula	0	0	0	0	0	0	0,029967036	0	0,019603999	0,072411296	0	0,127754711	0	0	0
Rhynchogastrema	0	0	0,214285714	0,237812128	0	0	0,089901109	0	0,137227995	0	0,015100038	0	0	0	0
Saccharomycopsis	0	0	0	0	0	0	0	0	0	0	0	0	0,143266476	0	0
Saturnispora	0	0	0	0	0	0,368300737	0	0	0	0	0	0	0	0	0
Sporobolomyces	0	0,048614487	0	0	0	0	0	0,040096231	0	0	0	0	0	0,196232339	0
Stagonosporopsis	0	0	0	0,118906064	0,122299225	0,025400051	0,029967036	0,100240577	0	0	0	0	0	0	0
Starmerella	0,921658986	0,194457948	0	0	0	0	0,329637399	0	0	0	0	1,053976365	0	0	0
Sterigmatomyces	0	0	0	0	0	0	0	0	0	0,036205648	0	0	0	0	0
Talaromyces	0,057603687	0	0	0	0	0	0	0	0	0	0,007550019	0	0	0	0
Trichoderma	0	0	0	0	0	0	0	0	0	0	0	0	0,015918497	0	0
Trichosporon	0	0,048614487	0	0	0,020383204	0	0	0	0	0	0,007550019	0	0	0	0

Table S2: Statistical analysis of glucose consumption

Glucose												
**Samples**	**A**	**B**	**C**	**D**	**E**	**F**	**G**	**H**	**I**	**J**	**K**	**L**
**T72**	****											
**T60**	****	****										
**M60**	****	****										
**M72**	****	****										
**T48**	****	****	****									
**M48**	****	****	****									
**T36**	****	****	****	****								
**T30**	****	****	****	****	****							
**M36**	****	****	****	****	****	****						
**M30**	****	****	****	****	****	****	****					
**T24**		****	****	****	****	****	****	****				
**T0**		****	****	****	****	****	****	****				
**T18**			****	****	****	****	****	****	****			
**M24**			****	****	****	****	****	****	****			
**T12**			****	****	****	****	****	****	****			
**B48**				****	****	****	****	****	****			
**B60**					****	****	****	****	****			
**T60**					****	****	****	****	****			
**B72**					****	****	****	****	****			
**M18**					****	****	****	****	****			
**B30**						****	****	****	****			
**B24**							****	****	****			
**B36**								****	****	****		
**M6**									****	****		
**M12**									****	****		
**B18**										****	****	

**B12**											****	****
**B6**											****	****
**M0**											****	****
**B0**												****

Table S3: Statistical analysis of fructose consumption

**Fructose**										
**Samples**	**A**	**B**	**C**	**D**	**E**	**F**	**G**	**H**	**I**	**J**
**T72**	****									
**T60**	****	****								
**T36**	****	****	****							
**T24**		****	****							
**T30**			****							
**T48**			****	****						
**M60**			****	****						
**T0**			****	****						
**M48**			****	****	****					
**M30**			****	****	****					
**T12**			****	****	****					
**T18**			****	****	****					
**M72**			****	****	****					
**M36**			****	****	****					
**M24**				****	****	****				
**T60**					****	****	****			
**M18**						****	****			
**M12**							****	****		
**B30**							****	****	****	

**B48**							****	****	****	
**M0**							****	****	****	
**B24**							****	****	****	
**B60**								****	****	****
**B36**								****	****	****
**M6**								****	****	****
**B72**								****	****	****
**B18**									****	****
**B60**										****
**B12**										****
**B0**										****

Table S4: Statistical analysis of lactic acid production

**Lactic Acid**														
**Sample**	**A**	**B**	**C**	**D**	**E**	**F**	**G**	**H**	**I**	**J**	**K**	**L**	**M**	**N**
**T6**	****													
**M6**	****	****												
**T12**	****	****												
**B6**	****	****	****											
**M12**	****	****	****	****										
**B12**		****	****	****										
**T24**		****	****	****	****									
**T18**			****	****	****	****								
**T30**				****	****	****								
**T36**				****	****	****								
**M18**					****	****	****							
**M24**						****	****	****						

**T60**							****	****	****					
**B18**							****	****	****					
**B24**							****	****	****					
**T48**							****	****	****					
**M30**							****	****	****					
**T72**							****	****	****	****				
**M36**								****	****	****	****			
**B30**									****	****	****			
**B36**										****	****	****		
**M48**											****	****		
**B48**												****		
**M60**												****		
**B60**													****	
**M72**													****	****
**B72**														****

Table S5: Statistical analysis of acetic acid production

**Acetic acid**										
**Samples**	**A**	**B**	**C**	**D**	**E**	**F**	**G**	**H**	**I**	**J**
**T24**	****									
**T18**	****									
**M18**	****	****								
**T30**	****	****								
**B18**	****	****								
**T36**	****	****								
**M24**	****	****	****							
**T60**		****	****	****						

**T72**		****	****	****						
**B24**			****	****	****					
**M30**				****	****	****				
**T48**				****	****	****	****			
**M36**				****	****	****	****			
**B30**				****	****	****	****			
**B36**					****	****	****	****		
**M48**						****	****	****		
**B48**							****	****		
**M60**								****	****	
**B60**									****	****
**M72**										****
**B72**										****

Table S6: Statistical analysis of volatile compounds detected in the liquid fraction from fermentation of Brazilian coffee beans

Compounds	0 h			12 h			24 h			48 h			72 h		
	Top	Middle	Bottom	Top	Middle	Bottom	Top	Middle	Bottom	Top	Middle	Bottom	Top	Middle	Bottom
Higher alcohols															
Ethanol	15.62±3 .97AB	15.16 ± 0.83 AB	18.25 ± 4.07 A	10.56 ± 1.05 B	16.68 ± 2.75 AB	20.10 ± 2.13 AC	16.10 ± 2.07 AB	16.76 ± 0.64 AB	20.53 ± 1.06 AC	41.05 ± 5.00 E	19.87 ± 1.12 A	21.44 ± 4.44 AC	17.62 ± 1.82 AB	27.02 ± 1.28 CD	33.85 ± 2.63 D
Methanol	-	0.98 ± 0.24 AB	0.73 ± 0.00 A	0.92 ± 0.14 AB	0.99 ± 0.32 AB	0.98 ± 0.42AB	1.16 ± 0.29AB	0.86 ± 0.04 AB	1.35 ± 0.09 AB	0.91 ± 0.21 AB	0.73 ± 0.16 A	1.09 ± 0.16 AB	0.88 ± 0.24 AB	0.96 ± 0.07 AB	1.54 ± 0.38 B
2-Butanol	-	-	0.12 ± 0.02 A	-	0.10 ± 0.01	-	-	-	-	0.88 ± 0.10 B	-	-	-	-	-
1-Propanol, 2-methyl	0.31±0. 18ABC	0.42 ± 0.16 BCD	0.57 ± 0.19 CD	0.39 ± 0.03 ABCD	0.67 ± 0.09 D	0.66 ± 0.13 D	0.22 ± 0.03 AB	0.24 ± 0.02 AB	0.22 ± 0.05 AB	0.13 ± 0.04 A	0.28 ± 0.01 ABC	0.40 ± 0.04 ABCD	0.23 ± 0.05 AB	0.35 ± 0.01 ABC	0.56 ± 0.03 CD
1-Butanol, 3-methyl	0.40±0. 09A	0.63 ± 0.12 AB	0.77 ± 0.17 ABC	0.91 ± 0.17 BCD	1.21 ± 0.20 CDE	1.38 ± 0.35 E	0.59 ± 0.16 AB	0.58 ± 0.06 AB	0.64 ± 0.01 AB	0.48 ± 0.10 AB	0.74 ± 0.06 AB	0.83 ± 0.14 ABCD	0.64 ± 0.11 AB	0.92 ± 0.08 BCDE	1.27 ± 0.12 DE
1-Butanol	-	-	-	-	0.20 ± 0.01 A	0.17 ± 0.11 AB	-	-	-	-	0.12 ± 0.02 B	0.13 ± 0.02 B	0.11 ± 0.00 B	0.17 ± 0.04 AB	0.21 ± 0.02 A
1-Hexanol	0.65±0. 05 A	0.83 ± 0.11 AB	0.66 ± 0.18 A	1.08 ± 0.12 AB	0.91 ± 0.11 AB	1.04 ± 0.01 AB	0.87 ± 0.23 AB	1.28 ± 0.22 ABC	1.29 ± 0.16 ABC	1.26 ± 0.78 ABC	2.08 ± 0.04 D	1.87 ± 0.06 CD	1.57 ± 0.15 BCD	2.99 ± 0.12 E	4.20 ± 0.37 F
2-Heptanol	1.65±0. 55A	2.48 ± 0.72 AB	3.13 ± 0.26 ABC	4.35 ± 0.63 BCD	4.70 ± 0.71 BCD	5.58 ± 0.61 CDE	5.10 ± 1.12 BCDE	6.46 ± 0.25 DE	7.49 ± 0.54 E	6.24 ± 1.51 DE	10.86 ± 0.24 F	11.49 ± 2.00 F	7.5 ± 0.64 E	12.71 ± 0.50 F	20.08 ± 0.91 G
3-Ethyl-4-methylpentan-1-ol	0.36±0. 05A	0.49 ± 0.04 AB	0.56 ± 0.14 ABC	0.90 ± 0.10 BCD	0.84 ± 0.12 BCD	0.92 ± 0.03 BCD	0.79 ± 0.19 ABCD	0.94 ± 0.06 BCD	1.28 ± 0.07 CDE	0.80 ± 0.13 ABCD	1.47 ± 0.05 E	1.25 ± 0.39 DE	1.10 ± 0.25 DE	1.95 ± 0.09 F	2.47 ± 0.19 G
Benzyl alcohol	-	0.32 ± 0.01 A	0.32 ±0.06 A	0.32 ± 0.08 A	0.39 ± 0.02 A	0.54 ± 0.11 A	-	0.33 ± 0.00 A	0.47 ± 0.07 A	0.47 ± 0.06 A	1.34 ± 0.21 B	1.08 ± 0.12 B	1.00 ± 0.06 B	2.51 ± 0.20 C	3.45 ± 0.23 D
Phenylethyl Alcohol	-	0.38 ± 0.05 AB	0.48 ± 0.08	0.71 ± 0.07 ABC	1.03 ± 0.01	1.22 ± 0.07 BCD	0.20 ± 0.05 A	0.67 ± 0.14 ABC	1.25 ± 0.06 CD	1.07 ± 0.16 BCD	4.12 ± 0.08 E	3.05 ± 0.60 F	1.58 ± 0.16 D	6.60 ± 0.27 G	9.88 ± 0.74 H

			ABC		ABCD										
3-Hexen-1-ol	-	-	-	-	-	0.20 ± 0.02 AB	-	-	0.15 ± 0.01 A	-	0.19 ± 0.04 AB	0.25 ± 0.03 AB	0.23 ± 0.02 AB	0.31 ± 0.10 B	0.57 ± 0.04 C
1-Octen-3-ol	-	-	-	0.28 ± 0.02 A	0.24 ± 0.04 A	0.30 ± 0.05 A	0.35 ± 0.02 A	0.38 ± 0.08 A	0.48 ± 0.08 C	0.41 ± 0.04 AB	0.66 ± 0.01 BC	0.73 ± 0.15 C	0.44 ± 0.12 AB	0.85 ± 0.07 C	1.22 ± 0.13 D
3-Octanol	-	-	-	0.41 ± 0.05 AB	0.38 ± 0.02 A	0.35 ± 0.02 A	0.40 ± 0.14 A	0.50 ± 0.06 AB	0.56 ± 0.05 ABD	0.701 ± 0.04 BCD	0.81 ± 0.06 CD	0.91 ± 0.13 C	0.50 ± 0.08 AB	0.98 ± 0.08 C	1.45 ± 0.20 E
6-methylhept-5-en-2-ol	-	-	-	-	-	-	-	0.25 ± 0.02	-	-	-	-	-	-	-
1-Propanol	-	-	-	-	-	-	-	-	0.13 ± 0.01	-	-	-	-	-	-
6-Hepten-1-ol, 2-methyl	-	-	-	-	-	-	-	-	-	0.57 ± 0.07 A	1.11 ± 0.02 B	1.11 ± 0.11 B	0.64 ± 0.03 A	1.45 ± 0.26 B	2.90 ± 0.06 C
1-Pentanol	-	-	-	-	-	-	-	-	-	-	0.20 ± 0.00 A	0.24 ± 0.05 A	0.22 ± 0.05 A	0.33 ± 0.02 AB	0.41 ± 0.07 B
2-Pentanol	-	-	-	-	-	-	-	-	-	-	0.20 ± 0.02 AB	0.12 ± 0.02 A	0.17 ± 0.07 A	0.40 ± 0.10 B	0.36 ± 0.00 B
2-Buten-1-ol, 3-methyl	-	-	-	-	-	-	-	-	-	-	0.57 ± 0.10 A	0.77 ± 0.11 A	0.61 ± 0.06 A	1.38 ± 0.06 B	1.29 ± 0.07 B
1-Pentanol, 3-ethyl-4-methyl	-	-	-	-	-	-	-	-	-	-	-	-	0.12 ± 0.02 A	0.35 ± 0.09 B	0.59 ± 0.07 C
2-Heptanol, 6-methyl	-	-	-	-	-	-	-	-	-	-	-	-	-	0.33 ± 0.09	-
Ketones															
2-Propanone	-	-	0.23 ± 0.03	-	-	-	-	-	-	-	-	-	-	-	-
2,3-Butanedione	0.46±0.15A	0.21 ± 0.09 B	0.28 ± 0.04 Ab	0.25 ± 0.05 AB	0.28 ± 0.00 AB	-									
2-Pentanone, 3-methyl	-	0.16 ± 0.06	-	-	-	-	-	-	-	-	-	-	-	-	-

Acetoin	0.42±0.04A	0.26 ± 0.17 A	0.73 ± 0.10 B	-	-	-	-	-	-	-	-	-	-	-	-
2-Heptanone	0.52±0.10A	0.19 ± 0.04 B	0.38 ± 0.02 A	-	-	-									
2-Butanone	-	-	-	-	-	-	-	-	-	-	-	-	-	-	-
beta.-Damascenone	-	-	0.35 ± 0.05 A	-	-	-	-	-	-	0.50 ± 0.06 AB	0.98 ± 0.16 C	0.85 ± 0.14 BC	0.96 ± 0.02 BC	2.79 ± 0.27 D	2.78 ± 0.27 D
Esters															
Methyl acetate	0.27±0.01AB	0.18 ± 0.04 A	0.23 ± 0.02 A	0.51 ± 0.04 ABCD	0.69 ± 0.03 BCDE	0.84 ± 0.14 DE	1.05 ± 0.13 EFG	1.46 ± 0.20 GH	1.79 ± 0.27 H	0.39 ± 0.13 ABC	1.32 ± 0.33 FG	1.01 ± 0.08 EF	0.55 ± 0.02 ABCD	0.72 ± 0.02 CDE	1.00 ± 0.12 EF
Ethyl Acetate	3.04±1.64ABC	2.16 ± 0.21 AB	1.69 ± 0.28 A	2.94 ± 0.66 ABC	4.80 ± 0.84 BCD	3.86 ± 0.85 ABC	7.69 ± 0.29 EF	7.44 ± 0.85 DEF	8.19 ± 0.31 F	16.39 ± 2.03 G	8.16 ± 1.04 F	5.65 ± 0.95 CDEF	18.37 ± 1.45 BC	4.50 ± 0.25 BC	5.02 ± 0.22 CDE
1-Butanol, 3-methyl-, acetate	-	-	-	-	-	-	-	0.28 ± 0.06 A	0.35 ± 0.04 BC	0.39 ± 0.14 ABC	0.52 ± 0.07 BC	0.49 ± 0.11 ABC	0.33 ± 0.07 AB	0.41 ± 0.06 ABC	0.59 ± 0.47 C
Acetic acid, 2-phenylethyl ester	-	-	-	-	-	-	-	-	-	0.47 ± 0.25 A	0.65 ± 0.05 A	0.55 ± 0.16 A	0.45 ± 0.09 A	1.24 ± 0.10 B	1.45 ± 0.15 B
Acetic acid, phenylmethyl ester													0.33 ± 0.06 A	0.64 ± 0.03 B	0.79 ± 0.01 C
Capric acid, ethyl ester	-	-	-	-	-	-	-		-	0.38 ± 0.10	-	-			
Isobutyl acetate	-	-	-	-	-	-	-	-	-	-	0.19 ± 0.00 A	0.18 ± 0.02 AB	0.07 ± 0.00 C	0.10 ± 0.03 C	0.12 ± 0.01 BC
Amyl acetate	-	-	-	-	-	-	-	-	-	-	0.15 ± 0.01 A	0.14 ± 0.01 A	-	0.16 ± 0.01	0.25 ± 0.03 B
2-Buten-1-ol, 3-methyl-, acetate	-	-	-	-	-	-	-	-	-	-	0.41 ± 0.02 A	0.21 ± 0.04 B	0.21 ± 0.02 B	0.67 ± 0.11 C	0.71 ± 0.01 C
Hexyl acetate	-	-	-	-	-	-	-	-	-	-	0.41 ± 0.03 AB	0.38 ± 0.15 AB	0.22 ± 0.01 A	0.51 ± 0.06 B	0.75 ± 0.03 C
Formic acid, 2-	-	-	-	-	-	-	-	-	-	-	0.65 ±	0.69 ±	0.36 ± 0.00	1.26 ±	1.52 ± 0.09

phenylethyl ester											0.06 A	0.13 A	B	0.05 C	D
Benzoic acid, 2-hydroxy-, methyl ester	-	-	-	-	-	-	-	-	-	-	1.00 ± 0.07 A	0.37 ± 0.02 C	1.12 ± 0.28 AB	1.48 ± 0.12 B	1.07 ± 0.12 AB
Propanoic acid, 2-hydroxy-, ethyl ester	-	-	-	-	-	-	-	-	-	-	-	-	-	0.17 ± 0.02	-
Free fatty acids															
Hexadecanoic acid, methyl ester	0.79±0.21AB	1.03 ± 0.24 ABCD	0.92 ± 0.28 ABC	0.87 ± 0.18 ABC	1.09 ± 0.25 ABCD	1.49 ± 0.08 D	1.23 ± 0.12 ABCD	1.04 ± 0.07 ABCD	1.26 ± 0.11 BCD	0.98 ± 0.19 ABCD	1.35 ± 0.08 CD	0.71 ± 0.06 A	0.98 ± 0.05ABCD	1.03 ± 0.05 ABCD	0.91 ± 0.15 ABC
Hexadecanoic acid, ethyl ester	-	5.02 ± 0.94 A	2.29 ± 0.70 B	2.81 ± 0.32 AB	2.75 ± 0.61 AB	3.56 ± 0.65 AB	2.54 ± 0.30 AB	2.13 ± 0.31 B	2.44 ± 0.14 B	5.10 ± 2.73 B	1.83 ± 0.09 B	1.15 ± 0.33 B	1.28 ± 0.34 B	1.09 ± 0.08 B	1.06 ± 0.06 B
Tetradecanoic acid, ethyl ester	0.40±0.04	-	-	-	-	-									
n-Hexadecanoic acid	4.58±1.49AB	6.23 ± 0.75 ABCDE	4.37 ± 1.11 A	5.78 ± 1.01 ABCD	5.37 ± 1.21 ABC	6.53 ± 1.43 ABCDE	6.99 ± 0.71 ABCDE	7.56 ± 1.44 ABCDE	9.21 ± 0.79 EF	7.77 ± 1.63 BCDEF	10.91 ± 0.20 AF	4.79 ± 0.27 AB	6.34 ± 0.77 ABCDE	8.95 ± 0.70 DEF	8.50 ± 1.54 CDEF
Tetradecanoic acid	-	0.37 ± 0.01 AB	0.33 ± 0.05 AB	0.30 ± 0.05 A	0.34 ± 0.02 AB	0.42 ± 0.07 AB	0.31 ± 0.05 AB	0.40 ± 0.07 AB	0.46 ± 0.07 ABC	0.47 ± 0.00 ABCD	0.68 ± 0.07 CDE	0.48 ± 0.05 ABCD	0.55 ± 0.19 BCDE	0.76 ± 0.13 E	0.71 ± 0.07 DE
n-Decanoic acid	-	-	-	0.14 ± 0.03 A	0.26 ± 0.04 B		-	0.17 ± 0.02 B	0.22 ± 0.02 BC						
Aldehyde															
Hexanal	1.69±0.30A	1.65 ± 0.30 A	1.96 ± 0.04 A	0.85 ± 0.03 CD	1.56 ± 0.30 AB	1.64 ± 0.04 A	1.16 ± 0.11 BC	0.72 ± 0.07 CDE	0.94 ± 0.13 CD	0.91 ± 0.08 CD	1.04 ± 0.03 CD	0.75 ± 0.05 CDE	0.67 ± 0.11 ED	0.76 ± 0.10 CDE	0.30 ± 0.05 E
Benzaldehyde	2.74±0.45ACD	2.93 ± 0.07 ABC	3.12 ± 0.07 AB	2.14 ± 0.02 DEF	2.68 ± 0.74 ACD	3.68 ±0.12 A	2.28 ± 0.18 CDE	1.62 ± 0.21 EFG	2.26 ± 0.13 CDE	1.10 ± 0.19 GH	1.58 ± 0.07 EFG	0.79 ± 0.05 H	1.41 ± 0.09 FGH	1.46 ± 0.07 FGH	1.15 ± 0.22 GH
2-Octenal	0.35±0.08ACD	0.39 ± 0.02 ABC	0.48 ± 0.08 AB	0.44 ± 0.08 AB	0.41 ± 0.07 AB	0.53 ± 0.09 B	0.18 ± 0.01 E	0.10 ± 0.00 E	0.24 ± 0.01 CDE	0.16 ± 0.01 E	0.19 ± 0.03 DE	0.13 ± 0.03 E	-	-	0.12 ± 0.02 E
Nonanal	0.37±0.	0.67 ± 0.19	0.34 ± 0.01	0.21 ±	0.33 ± 0.08	0.23 ± 0.04	0.23 ± 0.02	0.12 ± 0.03	0.57 ±	0.50 ± 0.05	0.32 ± 0.05	0.26 ±	0.15 ± 0.00	0.33 ±	0.52 ± 0.05

	08ACD	B	CDE	0.06 DEF	CDEF	DEF	DEF	F	0.00 AB	ABC	CDEF	0.23 ACD	EF	0.04 CDE	ABC
Decanal	0.19±0.03AB	-	-	-	-	-	-	-	-	-	0.21 ± 0.03 AB	0.15 ± 0.02 A	0.16 ± 0.07 A	0.30 ± 0.06 B	0.27 ± 0.03 AB
Benzaldehyde, 2,4-dimethyl	0.92±0.13A	0.88 ± 0.18 A	0.96 ± 0.27 A	1.49 ± 0.23 ABCD	0.91 ± 0.05 A	2.13 ± 1.05 CDE	2.84 ± 0.61 E	2.05 ± 0.25 BCDE	2.33 ± 0.46 DE	1.65 ± 0.16 ABCD	1.44 ± 0.03 ABCD	1.45 ± 0.19 ABCD	1.08 ± 0.12 ABC	1.43 ± 0.06 ABCD	1.79 ± 0.07 ABCDE
Pentadecanal	0.53±0.10ABC	1.44 ± 0.32 D	0.63 ± 0.13 BC	0.61 ± 0.03 ABC	0.32 ± 0.05 ABC	0.83 ± 0.40 C	0.11 ± 0.00 A	-	0.39 ± 0.09 ABC	0.27 ± 0.09 AB	0.18 ± 0.02 AB	-	-	-	-
Benzeneacetaldehyde	-	1.20 ± 0.09 A	1.12 ± 0.13 A	-	-	-	-	-	-	-	-	-	-	-	-
Butanal, 3-methyl	-	-	0.24 ± 0.04 A	0.08 ± 0.02 B	0.12 ± 0.02 B	0.14 ± 0.03 B	-	-	-	-	-	-	-	-	-
2-Hexenal	-	-	0.22 ± 0.03 A	0.08 ± 0.01 B	0.10 ± 0.01 BC	0.17 ± 0.05 AC	-	-	-	-	-	-	-	-	-
Hydrocarbon															
Benzene, 1,2,3-trimethyl	0.38±0.06A	-	-	0.26 ± 0.03 A	-	-	-	-	-	-	-	0.63 ± 0.08 B			
Benzene, 1,2,4-trimethyl	0.27±0.06A	-	-				1.04 ± 0.52 BC	0.17 ± 0.02 A	1.21 ± 0.00 B	0.36 ± 0.04 A	0.63 ± 0.09 AC	1.32 ± 0.00 B	0.37 ± 0.10 A	-	-
Benzene, 1-ethyl-2-methyl	-	-	-	-	-	-	0.25 ± 0.02 A	-	-	-	-	0.16 ± 0.02 B			
Heptadecane	0.19±0.03	-	-	-	-	-	-	-	-	-	-	-	-	-	-
Dodecane, 4,6-dimethyl	0.41±0.09A	0.42 ± 0.09 A	0.45 ± 0.09 A	0.50 ± 0.03 A	0.55 ± 0.11 A	0.50 ± 0.08 A	-	-	-	-	-	-	-	-	-
Mesitylene	1.24±0.23A	-	-	-	0.53 ± 0.07 B	0.51 ± 0.20 B	-	-	-	-	-	-	-	-	-
Nonadecane	-	0.36 ± 0.03	-				-	-	-	-	-	-	-	-	-
Acids															

Benzoic acid	-	-	-	-	0.47 ± 0.18	-	-	-	-	-	-	-	-	-	-
Butanoic acid	-	-	-	-	-	-	-	-	-	-	0.35 ± 0.04 A	0.30 ± 0.10 A		0.54 ± 0.01 A	
Terpenes	-	-	-	-	-	-	-	-	-	-	-	-	-	-	-
Linalool	2.04±0.32A	3.49 ± 0.43 A	3.23 ± 0.42 A	6.35 ± 1.23 AB	7.68 ± 1.37 AB	10.93 ± 0.39 B	11.60 ± 2.14 BC	18.14 ± 0.67 CD	21.89 ± 1.65 D	23.73 ± 4.31 D	35.05 ± 3.24 E	38.27 ± 5.41 E	22.42 ± 2.53 D	37.24 ± 1.02 E	63.06 ± 2.53 F
beta.-Myrcene	-	-	-	-	-	-	-	-	-	0.42 ± 0.07 A	0.51 ± 0.02 AB	0.67 ± 0.10 C	0.44 ± 0.03 AB	0.59 ± 0.03 BC	0.91 ± 0.02 D
D-Limonene	-	-	-	-	-	-	-	-	-	-					0.81 ± 0.07
alpha.-Terpineol	-	-	-	-	-	-	-	-	-	-	0.57 ± 0.06 A	0.50 ± 0.06 A	0.48 ± 0.09 A	0.24 ± 0.04 B	0.49 ± 0.09 A
trans-Linalool oxide (furanoid)	-	-	-	-	-	-	-	-	-	-	0.71 ± 0.07 A	0.73 ± 0.13 A	0.51 ± 0.07 A	1.06 ± 0.04 B	1.64 ± 0.17 C
Terpenes															
Linalool	2.04±0.32A	3.49 ± 0.43 A	3.23 ± 0.42 A	6.35 ± 1.23 AB	7.68 ± 1.37 AB	10.93 ± 0.39 B	11.60 ± 2.14 BC	18.14 ± 0.67 CD	21.89 ± 1.65 D	23.73 ± 4.31 D	35.05 ± 3.24 E	38.27 ± 5.41 E	22.42 ± 2.53 D	37.24 ± 1.02 E	63.06 ± 2.53 F
beta.-Myrcene	-	-	-	-	-	-	-	-	-	0.42 ± 0.07 A	0.51 ± 0.02 AB	0.67 ± 0.10 C	0.44 ± 0.03 AB	0.59 ± 0.03 BC	0.91 ± 0.02 D
D-Limonene	-	-	-	-	-	-	-	-	-	-					0.81 ± 0.07
alpha.-Terpineol	-	-	-	-	-	-	-	-	-	-	0.57 ± 0.06 A	0.50 ± 0.06 A	0.48 ± 0.09 A	0.24 ± 0.04 B	0.49 ± 0.09 A
trans-Linalool oxide (furanoid)	-	-	-	-	-	-	-	-	-	-	0.71 ± 0.07 A	0.73 ± 0.13 A	0.51 ± 0.07 A	1.06 ± 0.04 B	1.64 ± 0.17 C
Other															
3-ethyl-2-methyl, 1,3-Hexadiene	0.39±0.05A	0.53 ± 0.02 A	0.66 ± 0.23 A	-	-	-	-	-	-	-	-	-	-	-	-
Ethyl 9-hexadecenoate	1.37±0.41A	0.96 ± 0.18 ABC	0.47 ± 0.07 BC	0.30 ± 0.02 C	0.37 ± 0.07 C	0.40 ± 0.08 C	0.42 ± 0.05 C	0.31 ± 0.03 C	0.50 ± 0.12 BC	1.21 ± 0.64 AB	-	-	-	-	-

Nerol oxide	-	-	-	-	-	-	-	-	-	-	0.39 ± 0.07 A	0.42 ± 0.00 A	0.27 ± 0.01 A	0.84 ± 0.11 B	0.93 ± 0.11 B
Benzene, 1-methoxy-4-methyl	-	-	-	-	-	-	-	-	-	-	-	-	-	0.99 ± 0.10 A	0.88 ± 0.18 A
